# Super-resolution imaging reveals the evolution of higher-order chromatin folding in early carcinogenesis

**DOI:** 10.1038/s41467-020-15718-7

**Published:** 2020-04-20

**Authors:** Jianquan Xu, Hongqiang Ma, Hongbin Ma, Wei Jiang, Christopher A. Mela, Meihan Duan, Shimei Zhao, Chenxi Gao, Eun-Ryeong Hahm, Santana M. Lardo, Kris Troy, Ming Sun, Reet Pai, Donna B. Stolz, Lin Zhang, Shivendra Singh, Randall E. Brand, Douglas J. Hartman, Jing Hu, Sarah J. Hainer, Yang Liu

**Affiliations:** 1 Biomedical Optical Imaging Laboratory, Departments of Medicine and Bioengineering, University of Pittsburgh, Pittsburgh, PA 15213 USA; 2 Department of General Surgery, The First Affiliated Hospital of Dalian Medical University, Dalian, China; 3 Dalian Jinzhou First People’s Hospital, Dalian, China; 4 Department of Pathology, West China Second University Hospital, Sichuan University, 610041 Chengdu, China; 5 School of Medicine, Tsinghua University, No.1 Tsinghua Yuan, Haidian District, 100084 Beijing, China; 6 Department of Pathology, School of Medicine, Guangxi University of Science and Technology, Guangxi, China; 7 Department of Pharmacology and Chemical Biology, University of Pittsburgh, Pittsburgh, PA 15213 USA; 8 University of Pittsburgh Hillman Cancer Center, Pittsburgh, PA 15232 USA; 9 Department of Biological Sciences, University of Pittsburgh, Pittsburgh, PA 15213 USA; 10 Department of Cell Biology, University of Pittsburgh, Pittsburgh, PA 15213 USA; 11 Department of Pathology, University of Pittsburgh School of Medicine, Pittsburgh, PA 15213 USA; 12 Department of Medicine, Division of Gastroenterology, Hepatology and Nutrition, University of Pittsburgh, Pittsburgh, PA 15213 USA

**Keywords:** Nanoscale biophysics, Cancer epigenetics, Cancer imaging, Super-resolution microscopy

## Abstract

Genomic DNA is folded into a higher-order structure that regulates transcription and maintains genomic stability. Although progress has been made on understanding biochemical characteristics of epigenetic modifications in cancer, the in-situ higher-order folding of chromatin structure during malignant transformation remains largely unknown. Here, using optimized stochastic optical reconstruction microscopy (STORM) for pathological tissue (PathSTORM), we uncover a gradual decompaction and fragmentation of higher-order chromatin folding throughout all stages of carcinogenesis in multiple tumor types, and prior to tumor formation. Our integrated imaging, genomic, and transcriptomic analyses reveal functional consequences in enhanced transcription activities and impaired genomic stability. We also demonstrate the potential of imaging higher-order chromatin disruption to detect high-risk precursors that cannot be distinguished by conventional pathology. Taken together, our findings reveal gradual decompaction and fragmentation of higher-order chromatin structure as an enabling characteristic in early carcinogenesis to facilitate malignant transformation, which may improve cancer diagnosis, risk stratification, and prevention.

## Abstract (web summary)

Aberrant chromatin structure is often found in cancer. Here, the authors optimise super-resolution microscopy for pathological tissue and discovered a significant decompaction of chromatin folding in early carcinogenesis prior to tumour formation.

## Introduction

Aberrant chromatin structure visualized under conventional light microscopy is one of the most universal and striking characteristics in cancer cells^1^ and has long been used to diagnose cancer^2^. Despite its clinical significance, the molecular underpinning of aberrant chromatin structure in cancer cells remains elusive^3,4^. The fundamental unit of chromatin is nucleosome which is further packaged into higher-order structure, organized into condensed, transcriptionally repressed heterochromatin and open, transcriptionally active euchromatin domains. In particular, constitutive heterochromatin—the most condensed form of chromatin, enriched for trimethylated histone H3 lysine 9 (H3K9me3) and repetitive sequence—protects genomic integrity and stability^5^.

Many studies have found that defective heterochromatin results in genomic instability, an enabling characteristic for cells to acquire hallmarks of cancer^6^. Double knockout mice of H3K9 methyltransferases Suv39h1/2 exhibit chromosome instability and increased risk of tumor formation^7^. Large organized heterochromatin H3K9 modifications were substantially lost in cancer cell lines^8^ and in cells undergoing epithelial-to-mesenchymal transition^9^, a crucial mechanism in cancer development. Loss of heterochromatin reader protein HP1 causes chromosome segregation and is associated with cancer progression^10^. Depletion of a histone methyltransferase, G9a, results in the development of more aggressive tumors in mice that are genomically unstable^11^. At the genomic level, satellite repeats (enriched with H3K9me3) are required for heterochromatin formation and accurate chromosome segregation^12^.

Although the causal relationship between dysregulated heterochromatin function and increased genomic instability is a well-recognized mechanism to promote cancer progression, this phenotype was largely inferred by biochemical analysis of chromatin-associated proteins and DNA sequences. The underlying molecular-scale chromatin structure of dysregulated heterochromatin function in tumorigenesis remains largely unknown. This is due, in part, to the lack of sufficient resolution to visualize molecular-scale higher-order chromatin structure below the resolution limit of a conventional light microscope. Several important questions remain unanswered. What is the characteristic molecular-scale chromatin abnormality in cancer cells? Although coarse aggregation of heterochromatin has been a well-documented microscopic feature diagnostic of many cancers^2^, numerous biological studies reported the loss of heterochromatin-associated proteins and post-translational marks in cancer cells which cannot explain the aggregated heterochromatin structure^5,7,11^. Aberrant chromatin structure is a characteristic of cancer cells, but what happens in early carcinogenesis when cells still appear normal prior to tumor formation? Is the structural disruption a one-hit event or a gradual evolving process throughout carcinogenesis? Is there a common feature in chromatin structure underlying all stages of carcinogenesis in multiple tumor types? Answering these questions will have significant clinical implications to improve cancer risk stratification and ultimately early detection. For example, the high-risk precursors undergoing aggressive progression to cancer may exhibit distinct chromatin structure from those with low risk in precursors that are indistinguishable by pathologists.

Recent advances in super-resolution fluorescence microscopy has revolutionized biological imaging by overcoming the diffraction barrier that limits the resolution of conventional light microscopy to be ~250 nm ^13^. In particular, (direct)stochastic optical reconstruction microscopy [(d)STORM]^14,15^ has shown great promise in advancing our understanding of in situ nanoscale chromatin structure down to the level of nucleosome clusters (or nanodomains) at the scale of ~30 nm in cultured cells and small organisms (e.g., *Drosophila*)^16–20^. However, characteristic higher-order chromatin structure throughout tumorigenesis has not been fully characterized on pathological tissue, in part due to challenges in imaging pathological tissue such as autofluorescence, stronger scattering, and non-uniform background.

Here, we optimize a STORM-based super-resolution imaging method for robust and high-speed super-resolution imaging of higher-order chromatin structure on pathological tissue. Super-resolution imaging reveals significant fragmentation in DNA folding and heterochromatin decompaction in early stage of carcinogenesis even in normal-appearing tissue at risk for tumorigenesis prior to tumor formation. Integrated genomic and transcriptomic analyses also suggest more open chromatin structure and reveal disrupted heterochromatin occurred mostly in the satellite repeats of the genome with increased gene expression. We also observe enhanced transcription activities and impaired genomic stability as functional consequences after we disrupt heterochromatin structure. Furthermore, we find a gradual decompaction of heterochromatin structure throughout all stages of tumorigenesis, which is also a common feature from multiple tumor types. Finally, we show the potential of super-resolution imaging of heterochromatin structure to detect high-risk precursors that cannot be distinguished by conventional pathology. Together, our finding of heterochromatin decompaction at the nucleosome level underlies its importance in early carcinogenesis and opens a new avenue for improving cancer diagnosis, risk stratification, and facilitating the development and evaluation of new preventive strategies.

## Results

### PathSTORM

The super-resolved imaging capability of STORM is largely based on localization of sparsely distributed single fluorescent emitters at nanometer precision, and generally achieves best performance in thin and transparent cells. Stronger scattering and autofluorescence in tissue often produce high and non-uniform background that can significantly degrade image resolution and introduce artifacts^21^. We optimized STORM for imaging pathological tissue (referred to as PathSTORM), especially for formalin-fixed paraffin-embedded (FFPE) pathological tissue—the most common form of preserved pathological specimen. We obtained high-quality super-resolution images of higher-order chromatin structure on FFPE tissue section via the following three approaches: (1) optical clearing and index matching to reduce background, (2) extreme-value-based emitter recovery to correct residual heterogeneous background, and (3) a computationally effective localization method to improve localization precision of overlapping emitters^22^ (Supplementary Fig. 1).

We then validated the ability of PathSTORM to visualize higher-order chromatin structures at different epigenomic states in vivo in normal intestinal epithelial tissue (Supplementary Fig. 2). Super-resolution images of heterochromatin and euchromatin showed distinct and heterogeneous groups of nucleosome clusters at the scale of tens of nanometers, serving as the building blocks for higher-order chromatin structure. In particular, heterochromatin formed highly condensed large nanoclusters, while euchromatin exhibited more uniform or spatially diffuse nanoclusters, as indicated by the green arrows in Supplementary Fig. 2. These results were consistent with the previously reported higher-order chromatin structure on in vitro mammalian cell culture models^16,20^. We further confirmed the observed higher-order chromatin structure by comparing the reconstructed super-resolution images with those from ultra-thin frozen tissue sections (700 nm thick). The latter has been used to achieve low and uniform background with standard STORM imaging conditions^23^. As shown in Supplementary Fig. 3, the chromatin structure on FFPE tissue showed similar structural features as those from ultrathin tissue section. This result validated the ability of PathSTORM to achieve similar resolution of higher-order chromatin structures on pathological tissues compared to standard STORM.

### Disrupted higher-order chromatin folding in early carcinogenesis

Next, to visualize the changes of higher-order heterochromatin structure in carcinogenesis, we used a well-established mouse model of intestinal tumorigenesis—*Apc*^Min/+^ mice where a mutation in *adenomatous polyposis coli* (*Apc*) causes spontaneous development of multiple intestinal neoplasia (Min) and closely mimics familial adenomatous polyposis in human colorectal neoplasia^24^. Chromatin folding in the intestinal epithelial tissue was imaged using two complementary targets—H3K9me3 (a marker for heterochromatin that largely overlaps with the most condensed regions of DNA) and DNA (Supplementary Fig. 4).

**Fig. 1:**
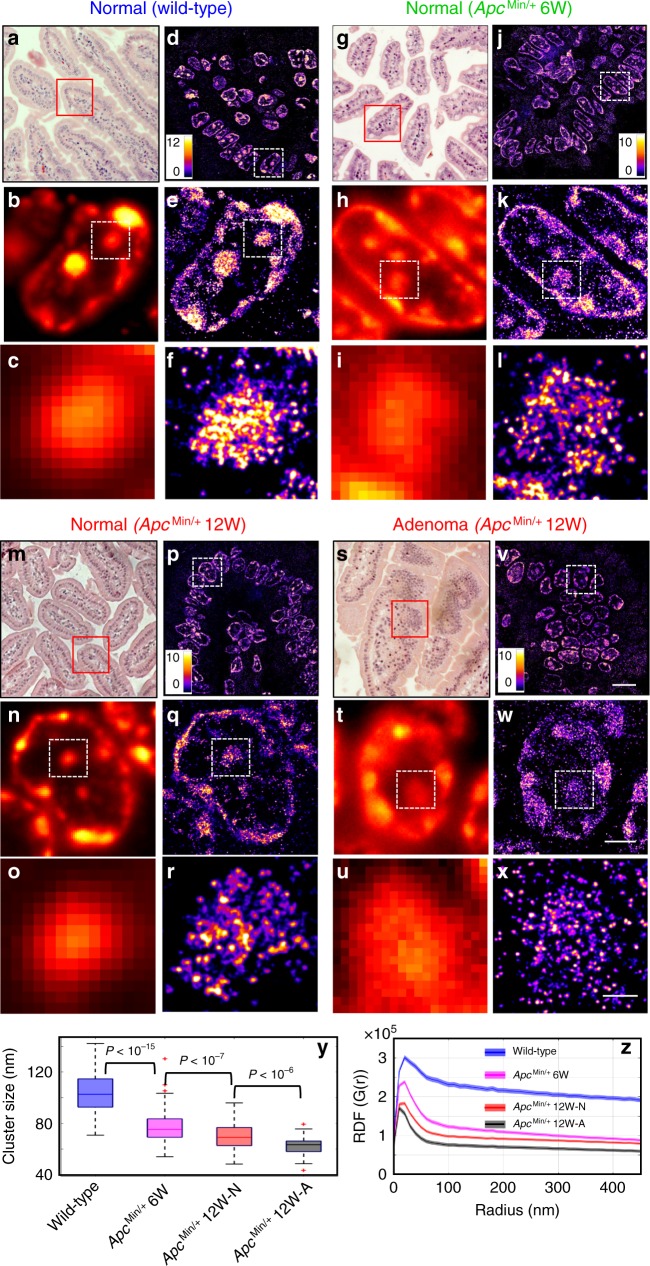
Super-resolution imaging of heterochromatin structure in *Apc*^Min/+^ mouse model. **a**, **g**, **m**, **s** H&E-stained pathology images and **d**, **j**, **p**, **v** STORM-based super-resolution images of H3K9me3-dependent heterochromatin from normal tissue from wild-type, histologically normal-appearing tissue from 6-week and 12-week *Apc*^Min/+^, and tumor (adenoma) from 12-week *Apc*^Min/+^ mice. Scale bar: 10 µm. **b**, **h**, **n**, **t** Conventional wide-field fluorescence images and **e**, **k**, **q**, **w** corresponding STORM images of heterochromatin from a single nucleus. (**c**, **i**, **o**, **u**) and (**f**, **l**, **r**, **x**) are progressively zoomed regions of (**b**, **h**, **n**, **t**) and (**e**, **k**, **q**, **w**). Scale bar in (**e**, **k**, **q**, **w**): 2 µm; scale bar in (**f**, **l**, **r**, **x**): 500 nm. **y** The box-and-whisker plots of the H3K9me3 cluster size, where the central line of the box indicates the median; the bottom/top edges of the box indicate 25th/75th percentiles; the whiskers extend to the most extreme data points without outliers from four groups. They include wild-type (*n* = 289 cells), normal-appearing tissue from 6-week (*n* = 259 cells) and 12-week *Apc*^Min/+^ (*n* = 99 cells) and tumor (adenoma) from 12-week *Apc*^Min/+^ mice (*n* = 55 cells). Cell nuclei from three mice for each category were analyzed. For each mouse, approximately five randomly selected areas of normal-appearing cells located in the villi regions were selected in wild-type and *Apc*^Min/+^ mice. In 12-week *Apc*^Min/+^ mice with tumors, we also analyzed those regions with adenoma and imaged 3−4 randomly selected tumor regions. **z** Average radial distribution function (RDF) for all nuclei in each group. The solid curve shows the average RDF from all measured nuclei and the shaded area shows the standard error. This definition was used throughout the entire manuscript. The *P* value for wild-type vs. 6-week *Apc*^Min/+^, for normal-appearing cells in 6-week *Apc*^Min/+^ vs. 12-week *Apc*^Min/+^, and for normal vs. tumor cells (adenoma) from 12-week *Apc*^Min/+^ mice are *P* < 10^−20^, *P* < 10^−9^ and *P* < 10^−3^, respectively. All *P* values were determined using Mann−Whitney test.

Figure 1 shows hematoxylin and eosin (H&E)-stained histology images (Fig. 1a, g, m, s), conventional wide-field fluorescence (Fig. 1b, c, h, i, n, o, t, u), and corresponding super-resolution images of heterochromatin in intestinal epithelial cells from villi regions, categorized in four groups—(a) normal intestinal epithelial cell nuclei of wild-type mice, (b) normal-appearing epithelial cell nuclei of intestinal mucosa at risk for tumorigenesis from 6-week *Apc*^Min/+^ mice without any visible tumor, (c) normal-appearing epithelial cell nuclei and (d) cell nuclei from adenoma in 12-week *Apc*^Min/+^ mice. The conventional wide-field fluorescence images (Fig. 1b, c, h, i, n, o, t, u) showed large and dense heterochromatin foci in all three types of tissue. In contrast, super-resolution images revealed that each of the large heterochromatin foci was formed by nucleosome nanoclusters. In normal cell nuclei from wild-type mice, nanoclusters were highly compact; in 6- and 12-week *Apc*^Min/+^ mice in early carcinogenesis when tissue still appeared normal, surprisingly, the nucleosome-level nanoclusters gradually became smaller and spatially segregated (Fig. 1j–l, p–r). Such structural disruption became even more severe in the tumor cells (adenoma) in 12-week *Apc*^Min/+^ mice (Fig. 1v–x). This result showed fragmentation of heterochromatin foci and nucleosome-level decompaction of higher-order heterochromatin structure. Such disruption in higher-order heterochromatin structure cannot be easily observed using conventional fluorescence microscopy. We also confirmed that the distinct heterochromatin structure between wild-type and *Apc*^Min/+^ mice was not due to the bias in resolution or localization density (see Supplementary Figs. 5, 6).

We then quantified the structural disruption of heterochromatin using two different methods—Gaussian mixed model clustering^25^ and radial distribution function (RDF) (a.k.a. pair-correlation function)^16,26^. The former quantified the size of nucleosome clusters (the building blocks that form the large heterochromatin foci); the latter provided a global overview for heterochromatin structure on multiple length scales. Figure 1y showed a progressive decrease in heterochromatin nanocluster size during carcinogenesis. The RDF distribution in Fig. 1z also showed a progressively narrower distribution and smaller correlation length, consistent with our observed progressive disruption of heterochromatin foci in Fig. 1a–x. Moreover, we did not observe a significant difference in normal epithelial cell nuclei between 6- and 12-week wild-type mice (Supplementary Fig. 7), suggesting that our observed higher-order structural disruption in heterochromatin was not due to age difference.

It should be noted that all normal-appearing cells from wild-type and *Apc*^Min/+^ mice in our analysis were differentiated intestinal epithelial cells located at the villi regions, confirmed by their characteristic morphology and negative Ki67 staining (Supplementary Fig. 8A). To further confirm the disrupted heterochromatin structure was not the effect of cell proliferation, we examined tumor cells (adenoma) from 12-week *Apc*^Min/+^ mice. As expected, some tumor cells showed positive Ki67 staining, indicating their proliferating status. But the heterochromatin structure on Ki67− and Ki67+ tumor cells did not show a significant difference (Supplementary Fig. 8C, E).

**Fig. 2:**
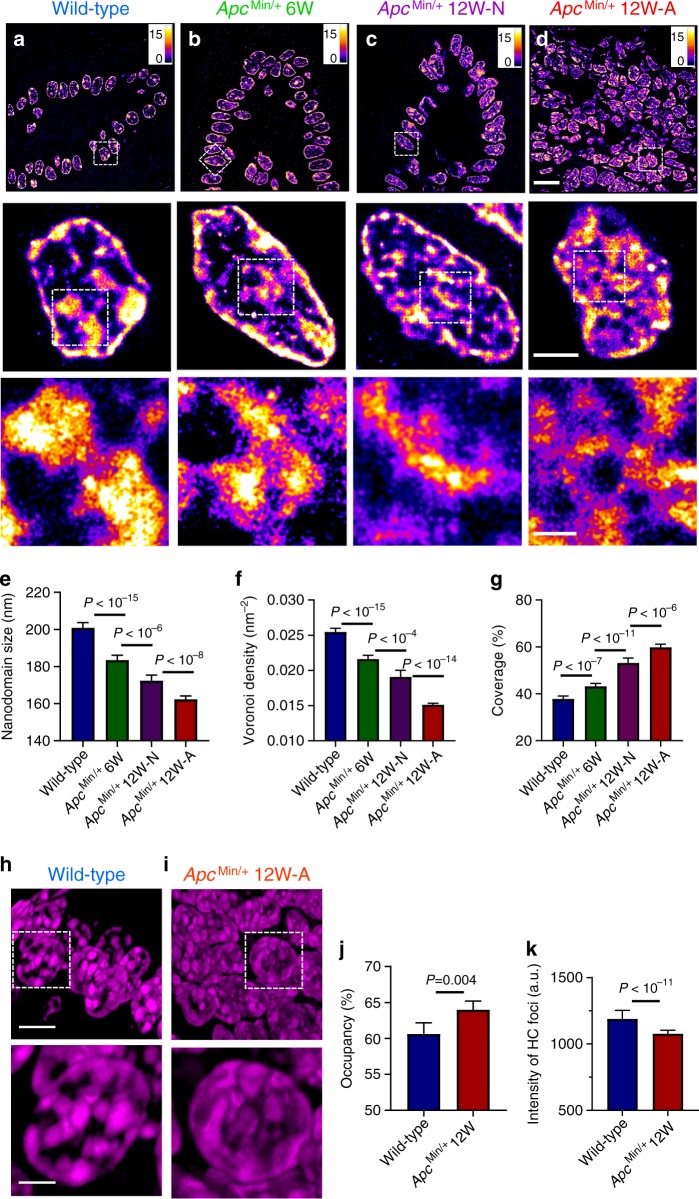
Super-resolution imaging of DNA in *Apc*^Min/+^ mouse model. **a**–**d** STORM images of DNA folding from normal cells from wild-type, histologically normal-appearing cells from 6-week and 12-week *Apc*^Min/+^, and tumor cells from 12-week *Apc*^Min/+^ mice. Scale bars: 10 µm, 2 µm and 500 nm in the original and magnified images, respectively. **e**–**g** The statistical analysis of DNA nanodomain size (**e**), the local density of DNA quantified by Voronoi polygon density (**f**) and percentage of occupied DNA domains for each nucleus (**g**) in normal cells from wild-type mice (*n* = 72 cells), from 6-week (*n* = 104 cells) and 12-week *Apc*^Min/+^ mice (*n* = 81 cells) and tumor cells from 12-week *Apc*^Min/+^ mice (224 cells). Error bars, mean ± 95% CI. Cell nuclei from three mice for each category were used in the above analysis, as described in the figure caption of Fig. 1. **h**, **i** 3D-SIM images of DAPI-stained DNA folding in normal cells from wild-type mice and tumor cells from 12-week *Apc*^Min/+^ mice. Scale bars: 5 and 2 µm in the original and magnified images, respectively. **j** The occupancy of DNA defined by the total volume of DNA over the entire 3D volume of each nucleus, calculated from the 3D-SIM images. **k** The average fluorescence intensity from each of the condensed region of heterochromatin (HC) foci in 3D-SIM images. Cells from wild-type mice (*n* = 33 cells) and tumor cells from 12-week Apc^Min/+^ mice (*n* = 61 cells) were used in the 3D-SIM image analysis. Error bars: mean ± 95% confidence interval (CI). All *P* values were determined using Mann−Whitney test.

We also performed super-resolution imaging on DNA labeled with a fluorophore (TOTO-3) that directly binds to nucleic acids. We validated the performance of TOTO-3 for STORM imaging of DNA compared to that labeled with the gold standard of Alexa 647 (Supplementary Fig. 9). As shown in Fig. 2a–d, STORM images of DNA also revealed progressively more fragmented and relaxed DNA folding from normal-appearing cells (intestinal epithelial cells from the villi, Ki67−, Supplementary Fig. 8B) from 6- and 12-week *Apc*^Min/+^ mice at risk for tumorigenesis compared to normal cells from wild-type mice. Similar to H3K9me3-marked heterochromatin, tumor cells from 12-week *Apc*^Min/+^ mice showed even more prominent fragmentation of disrupted DNA folding. To quantify their structural changes, we used watershed to calculate the size of the DNA nanodomains. We also quantified changes in chromatin compaction by the local density via Voronoi tessellation analysis^27,28^ (see Supplementary Fig. 10). Progressively reduced size of DNA nanodomains and lower Voronoi density were observed in normal-appearing cells from 6- and 12-week *Apc*^Min/+^ mice as well as tumor cells from 12-week *Apc*^Min/+^ mice (Fig. 2e, f) compared to cells from wild-type mice. The area of the nucleus covered by DNA also showed a significant increase along with tumorigenesis (Fig. 2g). The results from these quantitative image analyses collectively supported progressively fragmented DNA folding associated with chromatin decompaction. These results were consistent with the decompacted heterochromatin marked by H3K9me3. We also confirmed that the difference in higher-order chromatin structure between normal and tumor cells was not due to the difference in cell cycle in vitro (Supplementary Fig. 11) or cell proliferation in vivo (Supplementary Fig. 8C–F).

We further examined 3D chromatin structure (stained with DAPI) of normal cells from wild-type mice and tumor cells from 12-week *Apc*^Min/+^ mice using another super-resolution technique—3D structured illumination microscopy (3D-SIM) (Fig. 2h–i). As SIM has a lower resolution (~120 nm) compared to that of PathSTORM (~30 nm), the fragmented DNA nanodomains observed in PathSTORM cannot be easily seen in the 3D-SIM images. Instead, we observed more enlarged heterochromatin foci, supported by increased DNA occupancy (defined as the volume occupied by DNA over the entire 3D volume of the cell nucleus) in tumor cells (Fig. 2j). Further, as shown in Fig. 2k, the fluorescence intensity from the dense heterochromatin foci of tumor cells was significantly lower compared to that of normal cells from wild-type mice. The enlarged heterochromatin foci and their reduced fluorescence intensity collectively suggested structural decompaction. This observation of enlarged heterochromatin foci in tumor cells was largely in line with the common cytologic diagnostic criteria of “coarse aggregates of heterochromatin” in cancer cells observed under bright-field microscope^2^.

### Reduced H3K9me3 at regions with disrupted heterochromatin

**Fig. 3:**
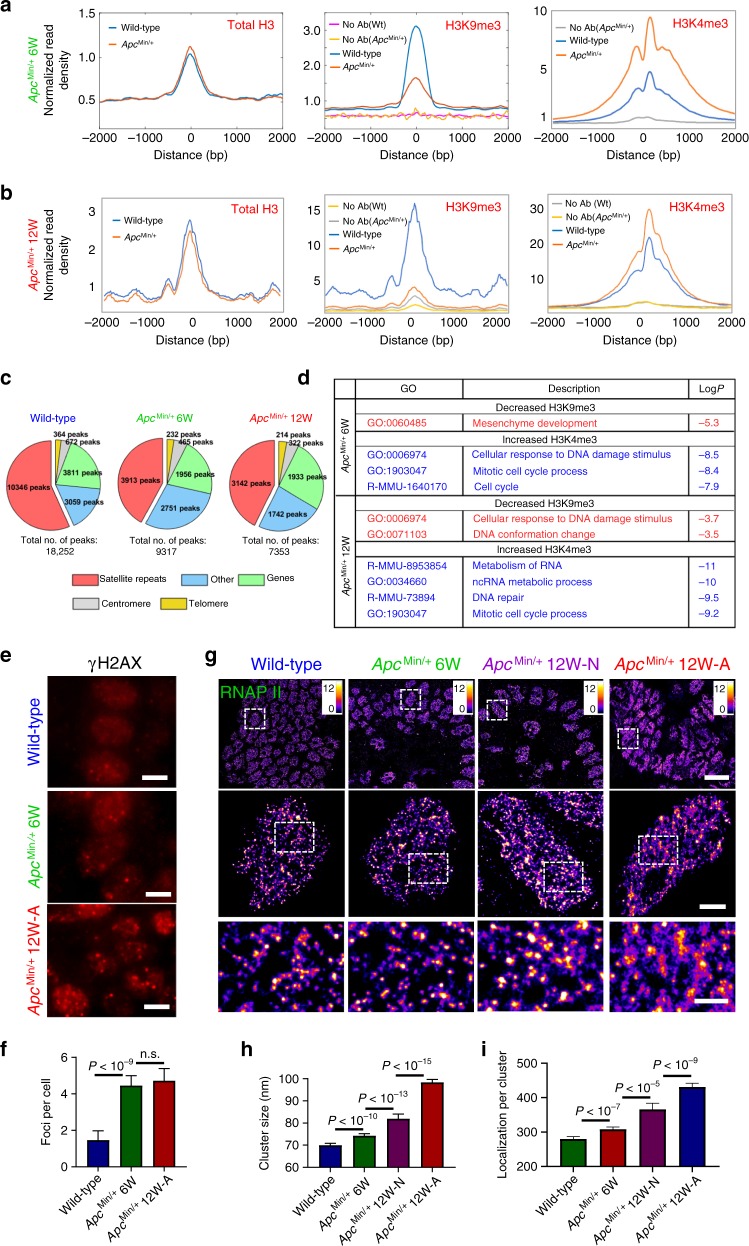
Impact of disrupted chromatin structure on transcription and genomic stability. **a**, **b** Average enrichment of H3K9me3 and total H3 from previously described H3K9me3 enriched genomic regions and average H3K4me3 occupancy over transcription start sites (TSSs), and **c** pie charts that show the genomic distribution of peaks that were enriched by H3K9me3, from normal-appearing intestinal tissue from wild-type mice and age-matched *Apc*^Min/+^ mice at 6 and 12 weeks, respectively. The genomic distribution in the pie chart consists of satellite repeats, genes, centromere, telomere and other genomic regions (including intergenic, other repeat regions that are unassigned to a type of repeat and non-annotated regions of the genome). **d** Gene ontology (GO) analysis for overlapping upregulated genes with reduced occupancy of H3K9me3 and increased occupancy of H3K4me3 of 6- and 12-week *Apc*^Min/+^ mice compared to age-matched wild-type mice. **e** γH2AX immunofluorescence staining in wild-type mice, *Apc*^Min/+^ mice at 6 and 12 weeks. Scale bars: 5 µm. **f** The number of γH2AX foci for each group (*n* = 50, 31, 28 cells, respectively). **g** The STORM images of active RNAP II from normal cells from wild-type, histologically normal-appearing cells from 6- and 12-week *Apc*^Min/+^ and tumor cells from 12-week *Apc*^Min/+^ mice. Scale bars: 10 µm, 2 µm and 500 nm in the original and magnified images, respectively. **h**, **i** Statistical analysis of the active RNAPII cluster size and number of localizations per cluster for each group (*n* = 301, 333, 174 and 321 cells, respectively). Cell nuclei from three mice were analyzed for each group, similar as described in Fig. 1. Error bars: mean ± 95% CI. All *P* values were determined using Mann−Whitney test.

To confirm our finding of disrupted heterochromatin structure in tumorigenesis and identify the affected genomic regions, we profiled native (no crosslinking) chromatin structure of intestinal epithelial cells brushed from normal-appearing mouse intestine of wild-type, age-matched *Apc*^Min/+^ mice at 6 and 12 weeks using CUT&RUN^29,30^. CUT&RUN is a genome-scale protein localization technique that serves as an alternative to chromatin immunoprecipitation (ChIP) but provides lower background signal. We profiled the occupancy of heterochromatin mark H3K9me3, euchromatin mark H3K4me3 and total histone H3, as well as a control lacking a primary antibody (but still including the proteinA-MNase to control for non-specific fragment digestion and release) referred to as “no antibody”. As shown in Fig. 3a, b, when we averaged the signal for H3K9me3 over previously described H3K9me3 genomic locations, we observed an enrichment of H3K9me3 relative to surrounding regions, while the “no antibody” control shows low background signal. Relative to wild-type mice (blue), we observed a substantial reduction in H3K9me3 occupancy in *Apc*^Min/+^ mice (red), while total H3 levels remain constant. The substantial reduction in H3K9me3 levels marking heterochromatin was accompanied by increased levels of H3K4me3 at promoter regions, suggesting more open chromatin.

As shown in the pie chart in Fig. 3c, the majority of H3K9me3 peaks identified in wild-type mice were within satellite repeats, as previously shown^31–33^. In comparison, in 6- and 12-week *Apc*^Min/+^ mice, the occupancy of H3K9me3 was significantly reduced, with less peaks identified overall (18,252 peaks in the wild-type (WT) mice, 9317 peaks in 6-week and 7353 peaks in 12-week *Apc*^Min/+^ mice), while the occupancy of total H3 protein remaining unchanged. The most significant reduction occurred at regions of satellite repeats (10,346 peaks in WT, 3913 peaks in 6-week and 3142 peaks in 12-week *Apc*^Min/+^ mice). These results not only support our imaging finding of disrupted heterochromatin structure (progressively reduced size of H3K9me3 nanoclusters) identified by PathSTORM, but also revealed that the disrupted heterochromatin mostly affects regions of satellite repeats in the genome of normal-appearing cells at early-stage carcinogenesis.

To explore the functional consequences of the disrupted heterochromatin structure, we performed genome-wide total RNA-seq on the same sets of mouse tissue. To identify the changes in gene expression directly altered by changes in H3K9me3 and H3K4me3 levels, we integrated CUT&RUN and RNA-seq analysis by assessing the gene expression changes of those genes that had reduced H3K9me3 or increased H3K4me3 in 6- and 12-week *Apc*^Min/+^ mice compared to wild-type mice. Out of the 1855 genes that had reduced H3K9me3 levels in 6-week *Apc*^Min/+^ mice, 820 genes showed a twofold or greater increase in gene expression (~44% of genes with H3K9me3 reduction). Importantly, the other 1004 genes that had reduced H3K9me3 did have modest increase in gene expression but did not reach the twofold cutoff. Of 6511 genes with increased expression in 6-week *Apc*^Min/+^ mice, we observed increased H3K4me3 at 6126 of these gene promoters. Similar trends were observed in 12-week *Apc*^Min/+^ mice. As shown in Fig. 2d, Gene Ontology (GO) enrichment analysis for 6-week *Apc*^Min/+^ mice identified that the most significantly enriched shared pathway in the gene sets associated with reduced H3K9me3 and with increased H3K4me3 was mesenchymal development (*P* < 10^−5^) (a process leading to acquisition of invasiveness and malignancy in epithelial cancer cells^9^) and cellular response to DNA damage stimulus and mitotic cell cycle process (*P* < 10^−8^) critical for maintaining genomic stability. The GO enrichment analysis for 12-week *Apc*^Min/+^ mice showed similar major pathways that also affected genomic instability (Supplementary Figs. 12, 13). These results collectively suggested that the disrupted heterochromatin structure in carcinogenesis was accompanied by more open chromatin that led to impaired genomic stability as the functional consequence.

### Impact on genomic stability and transcription

The above genomic and transcriptomic analyses suggest genomic instability and increased gene expression occur as a result of disrupted heterochromatin structure in early carcinogenesis. We examined these changes on the intestinal tissue in the *Apc*^Min/+^ mouse model. As shown in Fig. 3e, f, we observed a significantly increased number of γ-H2AX foci (*P* < 10^−9^)—a marker for DNA double-strand breaks—in *Apc*^Min/+^ mice, suggesting increased DNA damage or genomic instability. We further examined the phosphorylated form of RNA polymerase II (RNAPII)—a well-established maker for active transcription—with super-resolution microscopy, due to the well-documented association between RNAPII clustering and transcription activity^34–36^. As shown in Fig. 3g–i, we found a gradual increase in the cluster size of active RNA polymerase II (phosphorylated Ser5 of the RNAPII C-terminal domain, marking 5′ end of transcribing genes) and number of localizations per cluster from normal-appearing cells at risk for tumorigenesis (6- and 12-week *Apc*^Min/+^ mice) (*P* < 10^−10^ and *P* < 10^−13^, respectively), and tumor cells from 12-week *Apc*^Min/+^ mice (*P* < 10^−15^). These results suggested progressively increased transcription activities during tumorigenesis. We also validated the correlation between the active RNAPII cluster size and increased transcription activity by stimulating or inhibiting transcription using serum or DRB (5,6-dichloro-1-β-d ribofuranosylbenzimidazole) (Supplementary Fig. 14).

**Fig. 4:**
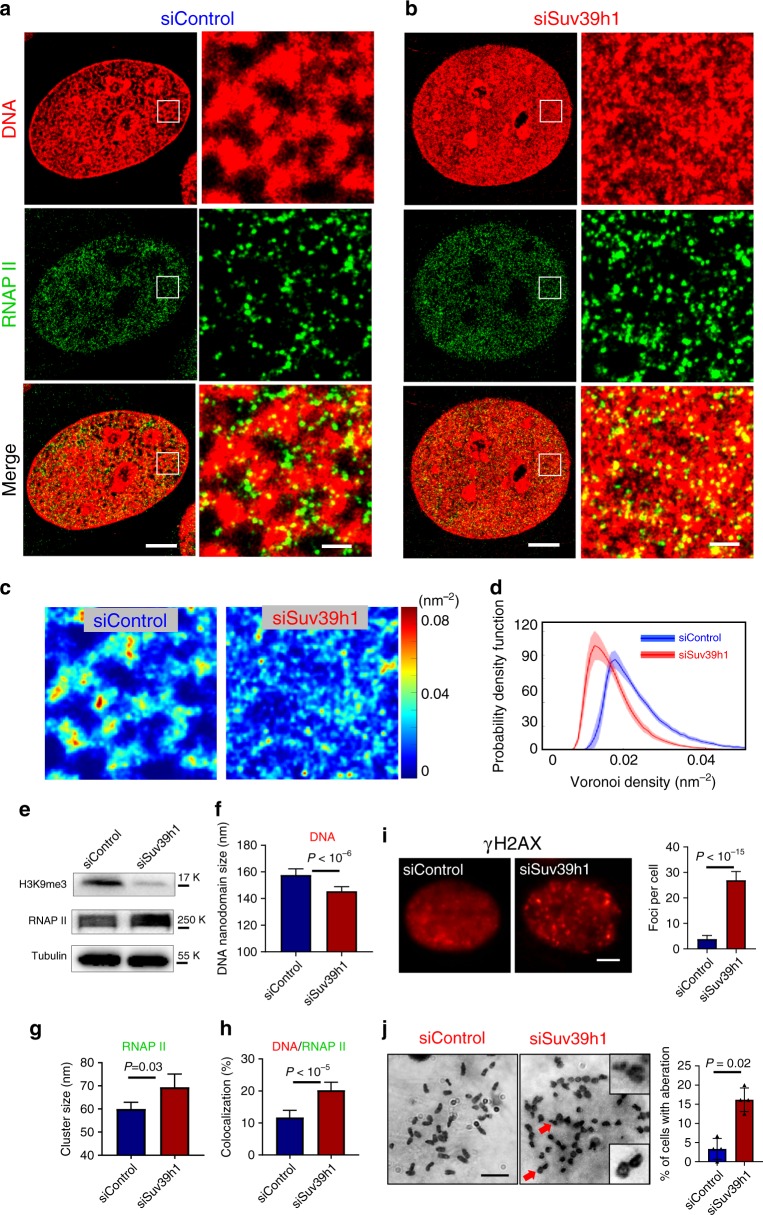
Functional and structural consequences upon disrupting heterochromatin structure. **a**, **b** Representative two-color STORM images showing the spatial relationship between DNA and active (phosphorylated) RNAP II in control NIH-3T3 cells and those cells with SUV39h1 knockdown (siSUV39h1). Green: phosphorylated RNAP II (labeled by Alexa 647); Red: DNA (labeled by CF568). Scale bars, 4 µm and 500 nm in the original and magnified images, respectively. **c** Local density map of DNA (quantified by Voronoi polygon density) of control and SUV39h1 knockdown cells. **d** Average histogram distribution of intra-nuclear local density of control and SUV39h1 knockdown cells. The solid curve shows the average RDF from all measured nuclei and the shaded area shows the standard error. **e** Western blotting of H3K9me3 and phosphorylated RNAP II with tubulin as an internal reference in control cells and those cells with SUV39h1 knockdown (siSUV39h1). **f**–**h** Quantitative analysis of DNA nanodomain size (*n* = 12 and 24 cells, respectively), active RNAP II (*n* = 24 and 24 cells, respectively) cluster size and the percentage of DNA that overlaps with active RNAP II (*n* = 14 and 27 cells, respectively) in control and cells with SUV39h1 knockdown. **i** γH2AX immunofluorescence and quantification of γH2AX foci numbers in control (*n* = 43 cells) and cells with SUV39h1 knockdown (*n* = 29 cells). Error bars: mean ± 95% CI. All *P* values were determined using Mann−Whitney test. **j** Cytogenetic analysis of chromosomal aberration in control cells or SUV39h1 knockdown cells. The enlarged regions showed chromosomes with breaks pointed by red arrows. Error bars: mean ± standard error, over 30 cells were counted per group in four randomly assigned groups. The full western blots are provided as Source data.

As H3K9me3 is placed by histone lysine methyltransferases, SUV39h is the master regulator of heterochromatin formation^37^. We analyzed the expression level of SUV39h1 in *Apc*^Min/+^ mice, which showed a significant reduction compared to wild-type mice (see Supplementary Fig. 15). In order to validate whether disrupting heterochromatin can result in fragmented DNA folding, increased transcription and impaired genomic stability, we knocked down SUV39h1 using small interfering RNA (siRNA) in NIH-3T3 cells. As shown in Fig. 4e, this depletion reduced the total level of H3K9me3 in mouse fibroblast cells and the knockdown efficiency was validated by western blot (Fig. 4e). The super-resolution images of H3K9me3 confirmed that upon depletion of SUV39h1, the large and compact heterochromatin foci in control cells became more disrupted with substantially reduced sizes of H3K9me3 nanoclusters (Supplementary Fig. 16). The super-resolution images of DNA (Fig. 4a, b) revealed more fragmented DNA folding in cells with SUV39h1 knockdown, as evidenced by the reduced size of nanodomains and reduced local density of DNA (Fig. 4c, d, f). The nanoclusters for the active RNAPII became enlarged in cells with disrupted heterochromatin and fragmented DNA folding (Fig. 4a, b, e, g), indicating enhanced transcription activities. Notably, upon disrupting heterochromatin structure in the siSUV39h1 cells, a significantly higher levels of decompacted heterochromatin domains were colocalized with active RNAPII (Fig. 4b, h and Supplementary Fig. 16D), suggesting that the decompacted chromatin domains were accompanied by active transcription. Our simulation also confirmed that such colocalization was not random (Supplementary Fig. 17). Further, we confirmed that cells with SUV39h1 knockdown showed an increased number of γ-H2AX foci and chromosomal instability (Fig. 4i, j). Together, these results suggested that disruption of heterochromatin structure indeed led to fragmented DNA folding, enhanced transcription activities and increased genomic instability.

### Disrupted heterochromatin structure in multiple tumor types

To evaluate whether such structural disruption is due to a specific cancer-driven molecular pathway or a common feature, we imaged heterochromatin structure in another mouse model of intestinal tumorigenesis—*Villin-Cre/BRAF*^V600E/+^ in which the tumor-initiating event in oncogene *BRAF* (mostly the mutation V600E) occurs in ~20% of colorectal carcinogenesis^38^. We quantified the cluster size and RDF of H3K9me3-dependent heterochromatin structure from normal cell nuclei of wild-type mice, non-dysplastic cells from *Villin-Cre/BRAF*^V600E/+^ mice at 6 weeks and tumor cells (adenoma) from *Villin-Cre/BRAF*^V600E/+^ mice at 12 months. As shown in Supplementary Fig. 18, the heterochromatin structure showed a substantial decompaction in early-stage carcinogenesis (6-week *Villin-Cre/BRAF*^V600E/+^ mice) when tissue appeared non-dysplastic and became more significant in tumor cells (12-month *Villin-Cre/BRAF*^V600E/+^ mice), as reflected in the progressively smaller H3K9me3 cluster size and narrower RDF distribution. These results suggested the disrupted heterochromatin structure in early carcinogenesis was independent of cancer-driven molecular pathways in intestinal tumorigenesis.

**Fig. 5:**
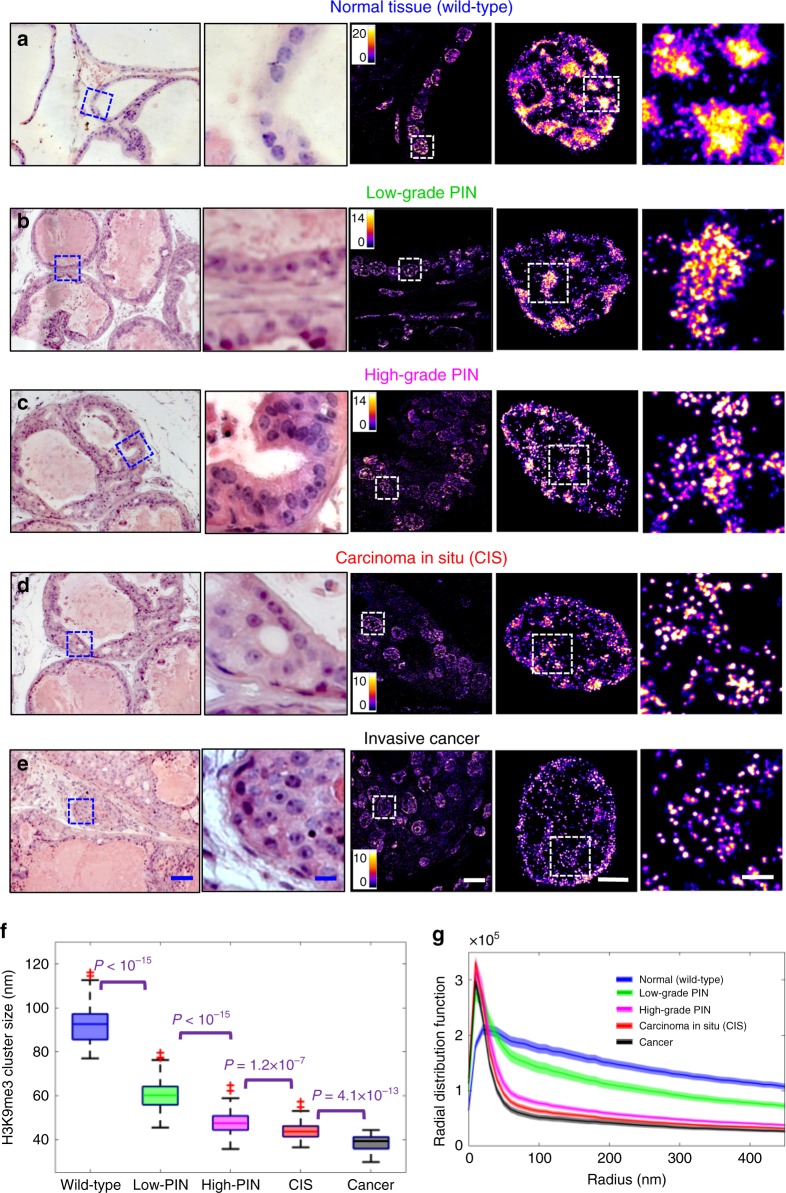
Super-resolution imaging of disrupted heterochromatin structure in prostate neoplasia. **a**–**e** Representative histology and the corresponding super-resolution images of heterochromatin structure (from the blue boxes) from normal epithelial cells of the prostate from wild-type mice, low-grade prostate intraepithelial neoplasia (low-grade PIN), high-grade PIN, carcinoma in situ (CIS) and invasive prostate carcinoma from Hi-*Myc* mice. Scale bars in the H&E images are 200 and 10 µm, respectively. Scale bars in the STORM images are 10 µm, 2 µm and 500 nm, respectively. **f** Box-and-whisker plot of the H3K9me3 cluster size (*n* = 111, 72, 110, 86, 69 cells, respectively). Cell nuclei from three mice for each category were analyzed. **g** Radial distribution function (RDF) that quantifies H3K9me3-dependent heterochromatin structure, averaged over all nuclei for each group. The shaded area shows the standard error. The *P* value for wild-type vs. low-grade PIN, for low-grade PIN vs. high-grade PIN, for high-grade PIN vs. CIS and for CIS vs. cancer is *P* < 10^−5^, *P* < 10^−8^, *P* < 0.05 and *P* < 0.05, respectively. All *P* values were determined using Mann−Whitney test.

We also used the *Myc*-driven prostate tumorigenesis mouse model (Hi-MYC), in which prostate cancer is driven by overexpression of Myc under the control of ARR_2_-probasin promoter^39^. This model is clinically relevant as *Myc* overexpression was reported in roughly 70% of early-stage prostate cancer^40–43^ and share molecular features with the human disease^39^. As shown in Fig. 5, we analyzed normal tissue from wild-type mice and a set of prostate lesions from Hi-MYC mice with low-grade prostate intraepithelial neoplasia (Low-PIN), high-grade PIN (high-PIN), carcinoma in situ (CIS), and invasive cancer. A similar progressive decompaction of heterochromatin in neoplastic progression of prostate lesions was observed, suggesting a gradual process throughout neoplastic progression (Fig. 5f, g).

Further, we used a fourth mouse model, one that generates pancreatic ductal neoplasm via pancreas-targeted expression of activated oncogene *Kras* (*Kras*^G12D/+^/*Pdx1-Cre*), and analyzed a spectrum of pancreatic lesions representing various stages of pancreatic neoplastic progression, including normal acinar cells from wild-type mice, acinar to ductal metaplasia (ADM) and pancreatic intraepithelial neoplasia (PanIN). In the *Kras* mouse model, acinar cells are the origin for PanIN lesions, and ADM is the initiating event for the development of pancreatic cancer^44–46^. As shown in Supplementary Fig. 19, similar to mouse models of intestinal tumorigenesis, we observed a significant decompaction of heterochromatin in the earliest precursor—ADM (Supplementary Fig. 19B); the size of heterochromatin nanoclusters undergoes progressive reduction with gradually narrower distribution of RDF during neoplastic transformation (normal acinar cells to ADM to PanIN). Therefore, the results shown here using three different tumor types supported that progressive decompaction of heterochromatin structure was a common feature across different tumor types throughout neoplastic progression.

### Disrupted heterochromatin structure in human neoplasia

**Fig. 6:**
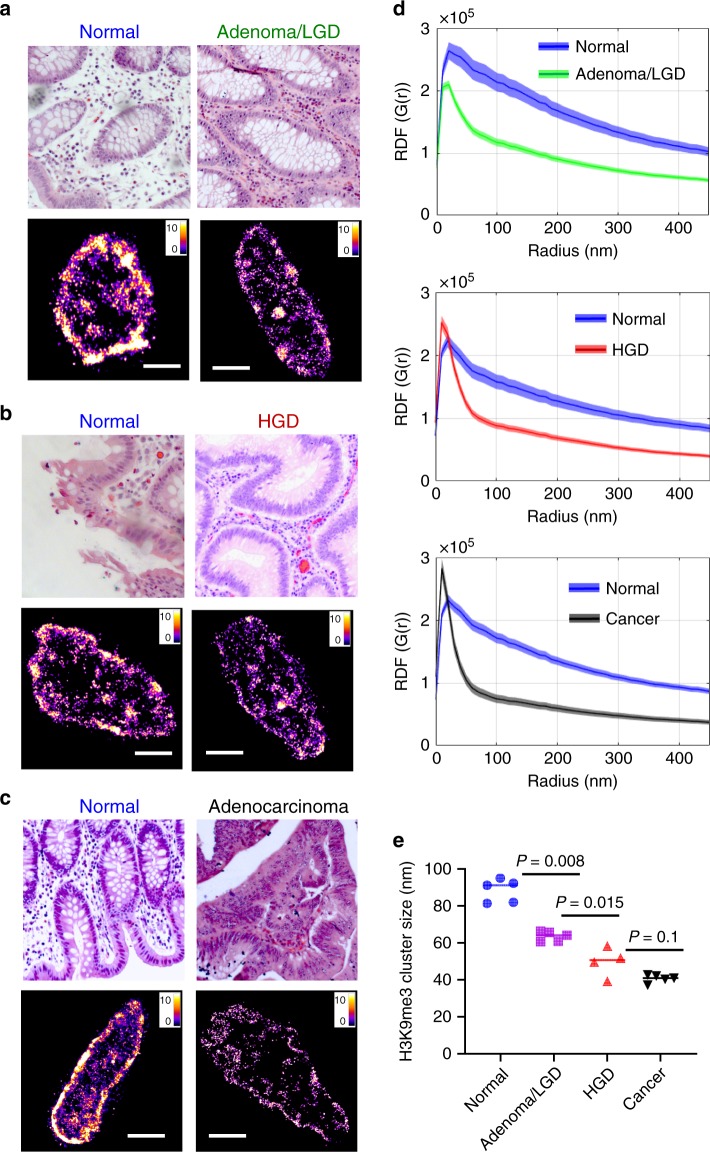
Super-resolution imaging of disrupted heterochromatin structure in human neoplasia. **a**–**c** Representative histology and corresponding super-resolution images of heterochromatin structure for colorectal neoplastic lesions together with their paired normal tissue located at more than 10 cm away from the tumor. Scale bars: 2 µm. **d** The radial distribution functions (RDF) for characterization of H3K9me3-dependent heterochromatin structure for the normal-tumor pairs. All *P* values between two groups are *P* < 10^−20^. **e** The average H3K9me3 cluster size for a total of 19 patient samples (patient information shown in Supplementary Table 1) including five normal tissue from non-neoplastic patients, five adenoma or low-grade dysplasia (LGD), four high-grade dysplasia and five invasive colorectal cancer. Each point is the average of over 100 cell nuclei for each patient. All *P* values were determined using Mann−Whitney test.

To confirm our mouse model findings in human tumors, we first analyzed a set of sporadic colorectal neoplasia and their paired normal tissue (Supplementary Table 1). As shown in Fig. 6a–d, both STORM images and the RDF show significant size reduction of heterochromatin nanoclusters in human tumors compared to the paired normal tissue. Figure 6e showed the results from 19 patient samples, and a progressive reduction in the size of H3K9me3 nanoclusters was also seen in normal tissue from non-neoplastic patients, adenoma or low-grade dysplasia (LGD), high-grade dysplasia (HGD), and adenocarcinoma (CA) with high significance. These findings in human neoplasia were consistent with our results in the above-described mouse models.

### Precursors indistinguishable by conventional pathology

**Fig. 7:**
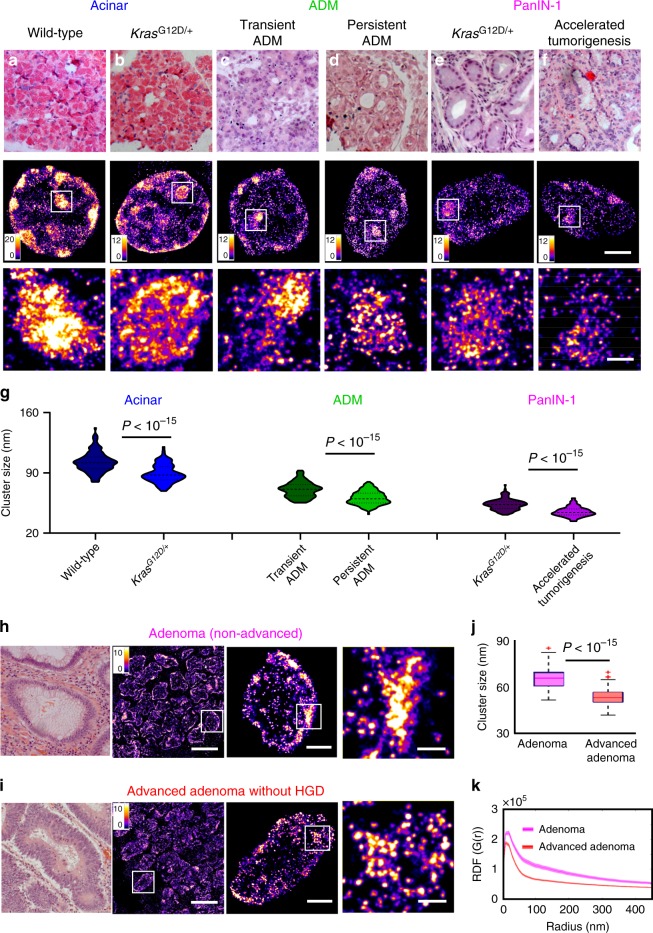
Disrupted heterochromatin structure in precursors indistinguishable by conventional pathology. **a**–**f** Representative histology and super-resolution images of heterochromatin structure in normal acinar cells between wild-type mice and *Kras*^G12D/+^ mice, between transient ADM from wild-type mice treated with cerulein and persistent ADM from *Kras*^G12D/+^ mice treated with cerulein, PanIN-1 lesions from *Kras*^G12D/+^ mice and *Kras*^G12D/+^ mice with cerulein treatment that underwent accelerated tumorigenesis. Scale bars in STORM images are 2 µm and 500 nm in the original and magnified images, respectively. **g** Statistical analysis of the H3K9me3 cluster size for each group. Acinar cell from wild-type (*n* = 115 cells) and *Kras*^G12D/+^ (*n* = 103 cells), transient ADM (*n* = 103 cells) and persistent ADM (*n* = 145 cells), *Kras*^G12D/+^ (*n* = 133 cells) and accelerated tumorigenesis (*n* = 101 cells). Data are presented as violin plots. **h**, **i** Representative histology and corresponding super-resolution images of heterochromatin structure between non-advanced adenoma and advanced adenoma without high-grade dysplasia. Note that adenoma and advanced adenoma without HGD are histologically indistinguishable, and advanced adenoma without HGD refers to those large adenoma with a tumor size of more than 1 cm (1.5 cm polyps). Scale bars in STORM images are 10 µm, 2 µm and 500 nm in the original and magnified images, respectively. **j** The box-and-whisker plots of the cluster size of H3K9me3-dependent heterochromatin (*n* = 105 and 154 cells, respectively). **k** The average radial distribution function (RDF) for H3K9me3, where the solid curve shows the average value from all measured nuclei and the shaded area shows the standard error. The *P* value between non-advanced adenoma and advanced adenoma without HGD is *P* < 10^−15^. *P* values were determined using Mann−Whitney test.

To explore the potential of nanoscale heterochromatin structure to detect high-risk precursors that cannot be distinguished by conventional pathology, we generated a more aggressive phenotype with accelerated neoplastic progression in the *Kras*^G12D/+^ mouse model of pancreatic tumorigenesis via inflammatory injury (treating mice with cerulein), which was compared to the *Kras*^G12D/+^ mice, as well as wild-type mice with and without cerulein treatment. First, we compared the STORM images of normal acinar cells in the pancreas of wild-type mice and *Kras*^G12D/+^ mice (Fig. 7a, b). Although acinar cells were normal phenotype, the acinar cells in *Kras*^G12D/+^ mice exhibited heterochromatin decompaction, as evidenced by smaller heterochromatin nanoclusters. Next, wild-type mice with cerulein treatment developed transient ADM, while *Kras*^G12D/+^ mice developed persistent ADM that eventually progressed into PanIN lesions. As shown in Fig. 7c, d, we found that the cell nuclei of persistent ADM exhibited more decompact heterochromatin compared to those of transient ADM. Further, as shown in Fig. 7e, f, the cell nuclei from PanIN-1 lesion developed from the cerulein*-*treated *Kras*^G12D/+^ mice that underwent accelerated tumorigenesis exhibited more heterochromatin decompaction compared to the same histological entity of PanIN-1 from *Kras*^G12D/+^ mice in the absence of inflammatory insult.

In addition, we analyzed the human precursor lesions that are at higher risk for developing cancer. Figure 7h, i showed a comparison of the STORM images between non-advanced and advanced adenoma without high-grade dysplasia. These two precursors were histologically indistinguishable, but advanced adenoma presents tumor size of larger than 1 cm that has been clinically established with a higher risk for developing colorectal cancer. The super-resolution images of advanced adenoma indeed presented smaller heterochromatin nanoclusters (Fig. 7j) and narrower distribution in RDF (Fig. 7k), consistent with our findings of more decompacted heterochromatin structure further along neoplastic progression. These results demonstrated that early-stage precursor lesions undergoing more aggressive neoplastic transformation exhibit more decompact heterochromatin structure, which may offer a more sensitive approach to risk-stratify patients at higher risk for aggressive progression.

## Discussion

This work revealed the evolution of higher-order heterochromatin structure and DNA folding throughout all stages of tumorigenesis. These findings were made possible by our optimized STORM-based super-resolution imaging coupled with high-speed and high-fidelity image reconstruction for pathological tissue, referred to as PathSTORM. This study addressed several important unanswered questions in the role of higher-order chromatin folding in tumorigenesis. Super-resolution imaging revealed significant heterochromatin decompaction and fragmented DNA folding in early carcinogenesis prior to the presence of tumor cells (Figs. 1, 2). We cross-validated this finding with an independent biochemical technique, CUT&RUN, which also identified that the most affected genomic regions by disrupted heterochromatin were satellite repeats. Integrated analysis of CUT&RUN with RNA-seq data revealed disrupted heterochromatin was accompanied with open chromatin and gene misregulation at locations where chromatin structure was altered, and specific upregulation of genes in pathways associated with genomic instability. Disrupting heterochromatin structure led to fragmented DNA folding, increased chromosomal instability, DNA damage and increased transcription activities. These results provided strong evidence for heterochromatin decompaction as an early event in malignant transformation that resulted in increased genomic instability and active transcription for cells to gain plasticity that facilitates malignant transformation^47^.

Quantitative analysis of higher-order chromatin folding at various stages of carcinogenesis (i.e., normal cells, different stages of precursors, and invasive cancer) revealed progressive heterochromatin decompaction, suggesting that such structural disruption was a gradual process throughout tumorigenesis, rather than a one-time event. Importantly, we showed that structural disruption in heterochromatin in early carcinogenesis was a common feature in multiple tumor types (colorectal, pancreatic, and prostate). These results suggested the role of heterochromatin decompaction as an enabling environment that facilitates malignant transformation. Of note, this finding would not be possible without the preserved spatial context of tissue architecture and cell morphology on pathological tissue, which is essential to unambiguously define pathological stages in carcinogenesis, especially for precursor lesions.

Our observed higher-order heterochromatin decompaction is largely in line with previous functional studies of dysregulated heterochromatin in cancer. The loss of heterochromatin proteins or post-translational marks (e.g., H3K9me3, H3K9me2, and HP1) was widely reported in cancer cells and its functional consequences of impairing genomic stability, changing transcriptional programs, and promoting tumorigenesis and cancer progression^3,5,7,10,11,48,49^. While few studies have examined dysregulated heterochromatin in precursor lesions, our study suggested that disrupting the compact heterochromatin structure prior to tumor formation may serve as an initial barrier for malignant transformation. Interestingly, H3K9me3-dependent heterochromatin was shown to be a barrier for cell fate change or reprogramming from somatic cells into induced pluripotent stem cells, which to some extent resembles tumorigenesis^47,50^. Ground-state pluripotent stem cells were found to present less dense nucleosome clutches containing fewer nucleosomes compared to somatic cells, which were also identified via STORM imaging^20^. Our data suggested that tumor cells share similar trait of disrupted heterochromatin and open chromatin organization with stem cells.

Our results of heterochromatin decompaction also provided a molecular-scale explanation for the cytologic feature of chromatin organization in cancer cells widely used for cancer diagnosis. Coarse aggregates of heterochromatin was frequently observed in cancer cells under conventional light microscope^2,51^ and even with electron microscope^52^. The aggregated heterochromatin may first appear contradictory to our observed heterochromatin decompaction and other biochemical studies of heterochromatin loss, but conventional cytology cannot distinguish these two processes. Due to the lack of molecular-scale resolution and quantitative ability, both features appeared as enlarged heterochromatin foci under a conventional light microscope. But super-resolution microscopy revealed fragmented DNA folding, reduced size of nucleosome nanoclusters, and less DNA molecules at the foci together with the increased occupancy of heterochromatin that collectively suggested structural decompaction rather than aggregation.

**Fig. 8:**
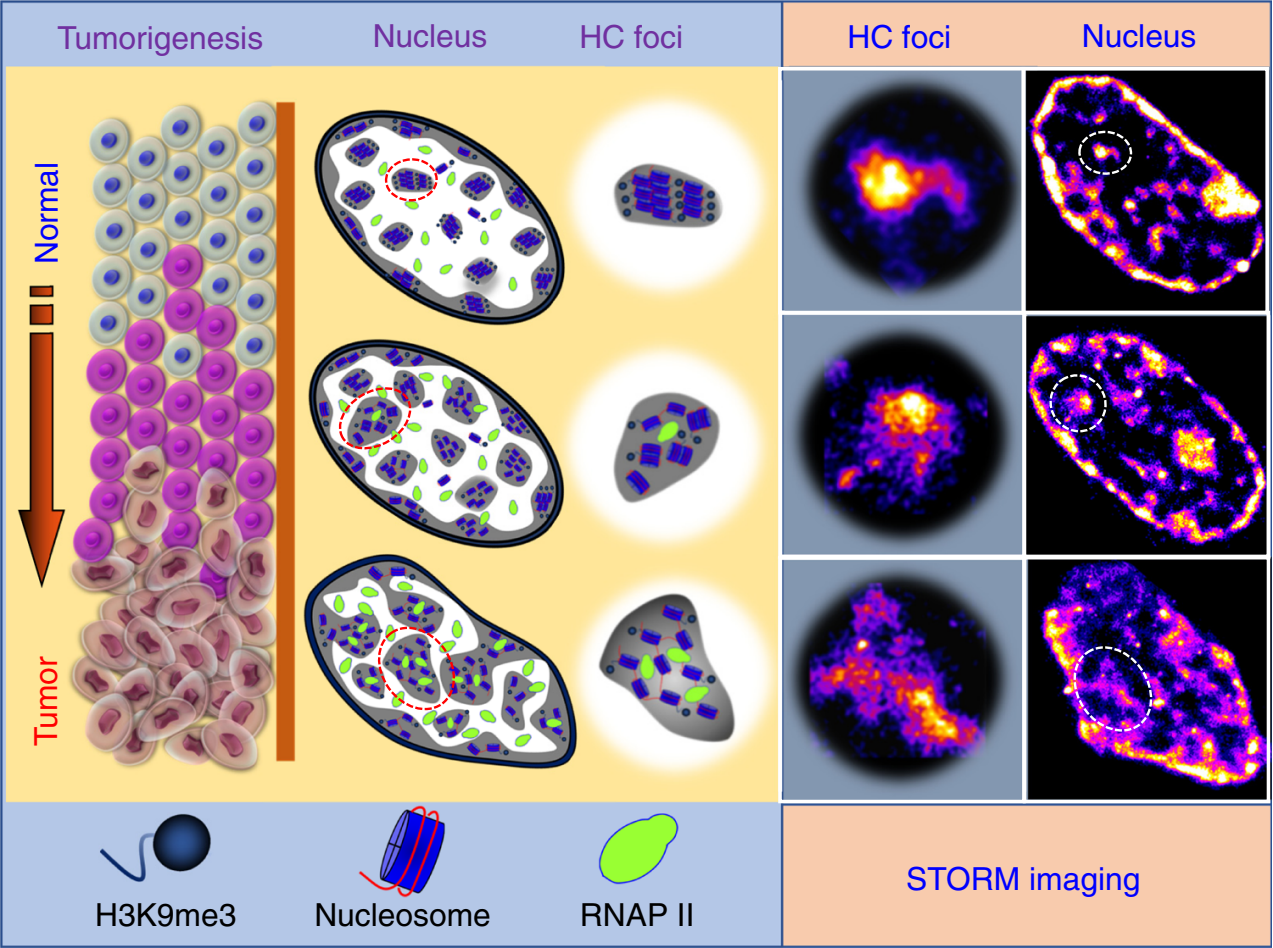
Model to depict the molecular-scale heterochromatin structure in carcinogenesis. As normal cells transform into tumor cells in carcinogenesis, chromatin folding becomes gradually de-compacted and fragmented accompanied with enlarged heterochromatin foci, which increases enhanced formation of transcription factories and genomic instability.

Our results suggest a model for the evolution of higher-order heterochromatin structure and DNA folding during malignant transformation from normal to cancer cells, as illustrated in Fig. 8. In normal cells, heterochromatin is highly compacted and spatially segregated from active transcription factories, protecting the cells from environmental insults. In early carcinogenesis when cells still appear normal, the heterochromatin mark (H3K9me3) undergoes a significant reduction, coupled with decompaction of the dense nucleosome clusters and fragmentation of DNA folding, leading to increased transcription activities coupled with genomic instability. When cells transform into tumor cells, chromatin folding becomes gradually decompacted and fragmented, leading to enlarged heterochromatin foci. Such gradual evolution of heterochromatin decompaction and fragmented DNA folding impair genomic stability and enhance transcription, which set proper environment for cells to gain plasticity and promote malignant transformation.

Ultimately, new scientific findings need to be translated to improve patient care. We explored the potential of imaging heterochromatin decompaction to improve cancer diagnosis and risk stratification. We showed that in those precursor lesions with the same pathological entity that were histologically indistinguishable, precursors at higher risk for accelerated malignant transformation presented more decompacted heterochromatin structure in both mouse models of accelerated tumorigenesis and human patients. For example, we showed that in ADM and PanIN-1 precursor lesions of pancreas, those that underwent accelerated tumorigenesis exhibited more decompacted heterochromatin structure. In adenoma and advanced adenoma without HGD (Fig. 7h–k) which were histologically indistinguishable, the heterochromatin exhibited more severe decompaction in advanced adenoma lesions compared to that from non-advanced adenoma. Although further validation on a large sample size is required, our data demonstrate the possibility of super-resolution imaging of higher-order heterochromatin structure to risk-stratify precancerous lesions.

Taken together, our study reveals the changes of nucleosome-level chromatin folding in malignant transformation, which shows gradual decompaction and fragmented DNA folding as a common feature independent of molecular pathways in multiple tumor types. Our results underline the importance of decompacted heterochromatin folding to facilitate malignant transformation in early carcinogenesis, which may also be used to improve cancer diagnosis, risk stratification, and facilitate the development and evaluation of new preventive strategies.

## Methods

### Immunofluorescence staining of tissue samples

All animal studies were performed in accordance with the institutional Animal Care and Use Committee at the University of Pittsburgh. All mice were housed in micro isolator cages in a temperature- (68–79 °F) and humidity (30–70% relatively humidity)-controlled room illuminated from 7:00 a.m. to 7:00 p.m. (12 h:12 h light–dark cycle), with access to water and chow ad libitum. The use of tissue blocks from deidentified patients was approved by the Institutional Review Board at University of Pittsburgh. FFPE tissue blocks taken from mouse models or patients were used in our study. The 3-µm-thick tissue sections mounted on the Poly-d-lysine (PDL)-coated coverslips were deparaffinized in xylene and rehydrated in graded ethanol and finally in distilled water. Cover glasses were then transferred to a preheated Tris-EDTA (Ethylenediaminetetraacetic acid) buffer solution (94–96 °C, containing 10 mM Tris Base, 1 mM EDTA, and 0.05% Tween 20, pH 9.0) and heated in a microwave for antigen retrieval. Sections were then washed in PBS (Phosphate buffered saline) and treated with 0.1% solution of sodium borohydride (Sigma-Aldrich) in PBS on ice to reduce autofluorescence. The sections were then permeabilized with 0.2% Triton X-100 (Sigma-Aldrich) in PBS. To block against non-specific binding, the sections were incubated with a blocking solution containing 3% BSA (Bovine serum albumin) and 0.2% Triton X-100 diluted in PBS for 1 h at room temperature. Then the sections were incubated with primary antibodies diluted to optimized concentrations at 4 °C overnight: rabbit polyclonal to H3K4me3 (1:300, ab8580, Abcam), rabbit polyclonal to H3K27me3 (1:300, Cat.07-449, Millipore), rabbit polyclonal to H4ac (1:200, Cat.06-598, Millipore), rabbit polyclonal to H3K9me3 (1:300, Cat.07-523, Millipore), mouse monoclonal to RNAP II (1:300, ab5408, abcam), mouse monoclonal to Ki67 (1:200, Cell Signaling Technology, #9449s), rabbit polyclonal to KMT1A/SUV39H1 antibody (1:100, Novus Biologicals, NBP2-17086) and mouse monoclonal to γ-H2AX (Ser 139) antibody (1:100, Santa Cruz Biotechnology, sc-517348). Then Alexa Fluor 647-conjugated donkey-anti-rabbit/mouse secondary antibody (1:200, Alexa Fluor 647 carboxylic acid succinimidyl ester, ThermoFisher, A20006; Donkey anti-rabbit antibody, Jackson ImmunoResearch, 711-005-152) was applied to the sections at room temperature for 2 h in the dark. Sections were then post-fixed in 4% paraformaldehyde for 10 min. The 60% of 2,2-thiodiethanol (TDE, Sigma-Aldrich) in PBS was used to optically clear the tissue sections for ~20–30 min. The fluorescent beads (FluoSpheres carboxylate, F8803, Thermo Fisher Scientific) were added into the sample dish as fiduciary markers used for drift correction based on our previously published method^25^.

### DNA staining in tissue section

The DNA from FFPE tissue sections was stained by TOTO™-3 Iodide (Thermo Fisher Scientific). The tissue section was first treated as described above before the blocking step and then treated with 50 µg/mL DNase-free RNase at 37 °C for 30 min. Then the tissue section was incubated with 100 nM TOTO-3 for 30 min at room temperature. After being washed three times in PBS, the sample was optically cleared as described above, now ready for STORM imaging. STORM imaging buffer for TOTO-3 stained tissue consists of 60% TDE (v/v), 10% (w/v) glucose, 0.56 mg/mL glucose oxidase and 0.17 mg/mL catalase.

### STORM image reconstruction

Prior to image reconstruction, the background correction based on extreme-value-based emitter recovery^22^ was applied, summarized as follows: (1) transform the intensity value from digital counts to physical photons; (2) segment it into a series of substacks (e.g. 100-frame where the background variation undergoes slow variation); (3) calculate the minimum value of each pixel along the temporal axis for each substack; and (4) calculate the expected background value according to our derived photon statistics model to obtain the expected background value and the temporal minima.

After background correction, we adopted a computationally efficient emitter-subtraction method, which is a variant of Gaussian deflation method used in high-density localization algorithms^53,54^ to restore the individual overlapped molecules by subtracting surrounding emitters without the computationally intensive iterative least squares fitting, followed by our previously developed single-iteration localization algorithm—gradient fitting^55^ for fast and precise single-molecule localization. All above procedures are simple mathematical operations, and thus can be efficiently implemented in GPUs for parallel processing. The reconstructed super-resolution image was rendered by accumulating all the valid molecules with a pixel size of 13 nm.

### Quantitative image analysis

Quantitative analysis of the reconstructed image was performed with a custom software written in MATLAB 2019 (Mathworks). We first segmented the epithelial cell nuclei using a semi-automated method and manual segmentation^16^. The localized point coordinates that form STORM images of H3K9me3 were clustered based on Gaussian mixed model (see Supplementary Fig. 20) to calculate cluster size and localizations per cluster^16^. RDF was determined by the density of localized spots as a function of distance to other localized spots for each nucleus^16^. For two-color STORM images, to correct for chromatic aberration, we used low-density multicolor fluorescence beads (TetraSpeck microspheres, 0.1 µm diameter, blue/green/orange/dark red fluorescence, Fisher Scientific) to generate the transform map^16^.

Voronoi tessellation was conducted for each nucleus, using the unique STORM coordinates as input arguments. The “voronoin” function in MATLAB R2019a was implemented, resulting in a set of region cells and diagram vertices which were used to calculate the area of each Voronoi polygon using “polyarea” function. Voronoi density was then calculated for each polygon as the inverse function of the polygon area. The values were then replotted at their corresponding point coordinates as a Voronoi density map^28,56^. The map was then smoothed using the Gaussian filter function “imgaussfilt” with a sigma of 2.

A Watershed segmentation algorithm was applied to the DNA density map, in order to segment the STORM points into localized groups or clusters^57^. The algorithm begins with the localization of the regional intensity maxima of the smoothed density map, conducted using “imregionalmax” function in MATLAB. The resultant binary map of maxima points was dilated using a disk-shaped kernel with a diameter of 5 pixels, then a distance transform of the inverse dilated map was calculated using the “bwdist” function. The resultant distance map was amplified by a factor of 2 before being added to a binary version of the smoothed density map. The density map was made binary by setting all values greater than zero to one. The watershed segmentation was then calculated on the negative of the added image and the segments outside of the binary density map region were rejected. Very small segments with a total pixel area less than 20 were also rejected from the final segmentation map. The examples of the watershed segmentation for the STORM images of DNA are shown in Supplementary Fig. 21.

To calculate the percentage of covered nuclei area by DNA, the reconstructed STORM image was first applied to a Gaussian filter (*σ* = 1) and the number of pixels with the value of greater than 0.5 divided by the total number of pixels for each nucleus mask is defined as the percentage of the covered nuclei area. The colocalization of DNA and active RNAPII was performed in two ways: the pixels in DNA that are overlapped with active RNAPII divided by the total number of pixels from DNA and the pixels in active RNAPII that are overlapped with DNA divided by the total number of pixels from active RNAPII, respectively.

### 3D-SIM image analysis

3D-SIM images were acquired by N-SIM system (Nikon). We performed 3D nucleus segmentation on the reconstructed 3D-SIM image via the following four steps: (1) Background was removed via a 3D median filter with a radius of 5 µm to estimate the local background caused by autofluorescence, which was subtracted from the SIM image. (2) To identify each 3D nucleus along the axial direction, we track the peak intensity distribution along the axial center position of the nucleus. (3) In the initial nucleus segmentation, at the axial position with the maximum intensity, the region of the nucleus was first manually selected by tracking the border of the nucleus, in which the central position was calculated. The precise border of each nucleus was then adaptively defined by finding the fastest changing gradient along each radial direction. This process was repeated for all axial positions above and below the central plane, for 3D nuclei segmentation. Finally, to calculate DNA occupancy, the voxels with an intensity larger than three times of square root of the background were recognized as the regions with valid signals. DNA occupancy was defined as the percentage of voxels with valid signals over the total number of voxels for each 3D nucleus.

### CUT&RUN

A total of eight mice including two *Apc*^Min/+^ mice (Jackson Laboratory), two age-matched wild-type mice at 6 weeks and two *Apc*^Min/+^ mice, two age-matched wild-type mice at 12 weeks were sacrificed, and their normal-appearing small intestine were removed and washed with PBS. After scraping villi cells from mouse intestine, cells were pelleted, washed with cold PBS, and nuclei were extracted in a hypotonic buffer (NE buffer; 20 mM HEPES-KOH pH 7.9, 10 mM KCl, 0.5 mM Spermidine, 0.1% TritonX-100, 20% glycerol, and freshly added protease inhibitors). CUT&RUN was performed as previously described^30^. Briefly, lectin-coated Concanavalin A beads (Polysciences) were prepared using binding buffer (20 mM HEPES-KOH pH 7.9, 10 mM KCl, 1 mM CaCl_2_, 1 mM MnCl_2_), and nuclei were bound to beads. Bead-bound nuclei were washed (wash buffer; 20 mM HEPES-KOH pH 7.5, 150 mM NaCl, 0.5 mM Spermidine, 0.1% BSA, and freshly added protease inhibitors) and primary (H3K9me3, abcam ab8898; H3K4me3, Millipore 05-745R; or total H3, abcam ab1791) was added to individual reactions. After a 2-h incubation at 4 °C with rotation and two washes in Wash Buffer, recombinant protein A-micrococcal nuclease (MNase) was added to identify the primary antibody. Following two washes in wash buffer, MNase cleavage was initiated with addition of 3 mM CaCl_2_, samples were incubated on a water-ice bath for 30 min and the reaction was chelated with addition of EDTA and EGTA (3,12-bis(carboxymethyl)-6,9-dioxa-3,12-diazatetradecane-1,14-dioic acid). RNAs were digested, and protected DNA fragments were released through centrifugation to separate solubilized DNA and protein from insoluble chromatin. Proteins were digested using Proteinase K, samples were purified through phenol chloroform extraction followed by chloroform extraction, and ethanol precipitated with glycogen. Libraries were prepared using Illumina protocols: end repair, A-tailing, and barcoded adapter ligation followed by PCR amplification and size selection. The integrity of the libraries was confirmed by quBit quantification, fragment analyzer size distribution assessment, and Sanger sequencing of ~10 fragments from each library. Libraries were sequenced using paired-end Illumina sequencing.

To analyze the data, reads were trimmed using Novocraft. Paired-end reads were aligned to mm10 using Bowtie2^58^ against the entire mouse genome (including repeat regions using RepeatMasker) with the parameters -N 1 and -X 1000. PCR duplicates were removed using Picard (http://broadinstitute.github.io/picard/) and reads with low quality score (MAPQ < 10) were removed using samtools. Reads sized 150–500 bp were separated for histone modifications. These reads were then processed in HOMER^59^. Reads were visualized at individual locations using the “makeUCSCfile” command. Mapped reads were aligned over major satellite regions of the genome using the “annotatePeaks” command to make 20 bp bins over regions of interest and sum the reads within each bin. Peaks were independently called using the “findPeaks” command, using the “no antibody” control for background signal, to identify all binding sites throughout the genome. Peaks were compared between control and experimental samples using the “mergePeaks” command. Motifs were identified using the “findMotifs” command.

### RNA-seq and transcriptomic analysis

Whole-genome strand-specific RNA-seq was used to profile RNA expression levels in intestinal cells from the same 6- and 12-week *Apc*^Min/+^ mice and age-matched wild-type mice used for CUT&RUN experimentation. RNA-Seq libraries were prepared as described previously^60^ and in the literature^61^. RNA was extracted from intestinal cells using TRIzol followed by column purification (Zymo RNA clean and concentrator column) following the manufacturers’ instructions. Total RNA was depleted of rRNA using a Ribo-Zero Gold kit and first-strand cDNA was synthesized. Subsequently, second-strand cDNA was synthesized, purified, and fragmented. RNA-seq libraries were prepared using Illumina technology, in a similar way as CUT&RUN libraries described above.

Paired-end reads were aligned to mm10 (mouse models) using RSEM^62^. We assessed the transcripts per million (TPM) changed and visualized the gene expression changes in the wild-type and *Apc*^Min/+^ mice. RSEM output files were used for downstream analysis using HOMER^59^. DESeq2 ^63^ was used to identify the differentially expressed genes based on significance. To sort the data, K-means clustering was performed using Cluster 3.0^64^ and heatmaps were generated using Java TreeView^65^. Gene Ontology (GO) term enrichment was performed to identify gene classes that are mis-regulated using Metascape software^66^. We integrated RNA-seq and CUT&RUN data to determine the direct effects of changes in heterochromatin structure on gene expression changes, in which the GO term analysis was performed to identify the direct target molecular pathways due to disrupted heterochromatin structure.

### Statistics and reproducibility

All statistical comparisons were made between two groups, calculated using non-parametric Mann–Whitney *U* test in GraphPad Prism 7.0 and two-tailed *P* value at 95% confidence interval was presented throughout the manuscript. Animal experiments were repeated from three mice and all experiments with cell lines were repeated for more than three times with similar results. All imaging data—performed on approximately 10–300 cells—were quantified and statistically analyzed. The average cluster size and nearest neighbor distance (nnd) were calculated for each nucleus as a data point. In the box-and-whisker plot, the central line of the box indicates the median; the bottom/top edges of the box indicate 25th/75th percentiles; the whiskers extend to the most extreme data points without outliers throughout the manuscript.

### Reporting summary

Further information on research design is available in the Nature Research Reporting Summary linked to this article.

## Supplementary information

Supplementary Information

Reporting Summary

## Data Availability

All CUT&RUN and RNA-seq sequencing data are deposited at Gene Expression Omnibus with accession code GSE121800. Representative raw data of STORM imaging videos are located at: https://pitt.app.box.com/v/PathSTORM-RawData. All other relevant data are available within the Article, Supplementary Information files or from the corresponding authors upon reasonable request.

## Source data

Source Data

## Code availability

The codes for data analysis are available from the corresponding authors upon reasonable request.
