# Supplementary material for: Super-resolution imaging reveals the evolution of higher-order chromatin folding in early carcinogenesis
Source: Nat Commun. 2020 Apr 20;11:1899. doi: 10.1038/s41467-020-15718-7 (PMC7171144; doi:10.1038/s41467-020-15718-7)
Supplement: Supplementary file 1 — Supplementary Information [file 41467_2020_15718_MOESM1_ESM.pdf]

Supplementary Information

Super-resolution imaging reveals the evolution of higher-order chromatin folding in early carcinogenesis

Xu et al.

<u>Supplementary Methods</u>

Animal models

Apc$^{Min/+}$ ***mouse model of intestinal tumorigenesis***

A total of 12 mice were included in this study for PathSTORM. A first set of three wild-type mice (C57BL/6J, The Jackson Laboratory, Stock No 000664) and three age- and sex-matched *Apc*$^{\mathrm{Min/+}}$ mice (C57BL/6J-*Apc*^{Min}/J, The Jackson Laboratory, Stock No 002020) were sacrificed at 6 weeks of age. A second set of three *Apc*$^{\mathrm{Min/+}}$ mice were sacrificed at 12 weeks of age. The 6-week *Apc*$^{\mathrm{Min/+}}$ mice did not show any visible tumor or dysplasia as confirmed by the pathologist; whereas those 12-week *Apc*$^{\mathrm{Min/+}}$ mice had developed multiple visible adenomatous polyps and histologically visible dysplasia in their small intestine. Small intestine tissue was removed, washed with phosphate buffered saline, and prepared in bundles of 1 cm segments. We fixed the tissue in 10% neutral buffered formalin for over 24 hours and embedded the tissue in paraffin block. A segment of small intestine (both proximal and distal parts) was cut, and the tissue was placed in 10% neutral buffered saline for over 24 hours. Then the tissue was embedded in paraffin block.

Villin-Cre;LSL-BRAF$^{V600E/+}$ ***mouse model of intestinal tumorigenesis***

The *Villin-Cre;LSL-BRAF*$^{\mathrm{V600E/+}}$ mice (B6.129P2(Cg)-*Braf*^{tm1Mmcm}/J, The Jackson Laboratory, Stock No 17837) were generated by crossing *Villin-Cre* mice (B6.Cg-Tg(Vil1-cre)1000Gum/J, The Jackson Laboratory, Stock No 21504) with *LSL-BRAF*$^{\mathrm{V600E/+}}$ mice which were obtained from the Jackson Laboratory (Bar Harbor, ME). Genotyping was performed according to protocols described by the Jackson Laboratory. The *Villin-Cre;LSL-BRAF*$^{\mathrm{V600E/+}}$ mice were euthanized at 6 weeks and 1 year of age. Small intestinal tracts were carefully dissected, rinsed with ice-cold saline, fixed in 10% neutral buffered formalin overnight, and further embedded in paraffin. Small intestine from 6-week-old *Villin-Cre* mice were used as controls.

Hi-Myc mouse model of prostate tumorigenesis

Male FVB-Tg(ARR2/Pbsn-MYC) mice were procured from the NIH mouse repository (STRAIN 01XK8) and bred in-house following animal protocol approved by the Institutional Animal Care and Use Committee at the University of Pittsburgh. After genetic verification by PCR, 3 pairs of mice (3 wild-type and 3 Hi-*Myc* mice) at 5 weeks of age were fed with AIN-93G diet and sacrificed at 26 weeks of age. Prostate tissues were collected at the time of sacrifice and fixed in 10% neutral buffered formalin and paraffin-embedded.

Pdx1-Cre;LSL-KRAS$^{G12D/+}$ ***mouse model of pancreatic tumorigenesis***

The *Pdx-Cre* (B6.FVB-Tg(Pdx1-cre)6Tuv/Nci, STRAIN 01XL5) and *LSL-KRAS*$^{\mathrm{G12D/+}}$ (B6.129-*Kras*^{tm4Tyj}/Nci, STRAIN 01XJ6) mice were received from the NCI Mouse Repository. These mice were crossed to generate *Pdx-Cre;LSL-KRAS*$^{\mathrm{G12D/+}}$ mice. Genotyping was performed according to the protocols

described by the NCI Mouse Repository. Three *Pdx-Cre;LSL-KRAS*$^{G12D/+}$ mice at the age of 7-month were euthanized, the pancreas was dissected, fixed overnight in 10% neutral buffered formalin, and embedded in paraffin. STORM imaging was performed in the pancreatic intraepithelial neoplasia (PanIN) lesions graded at PanIN-1 and PanIN-2. In addition, we treated three 6-week-old C57BL/6 mice (Jackson Laboratory, Bar Harbor, ME) and six age-matched *KRAS*$^{G12D/+}$ mice by six hourly intraperitoneal injection of caerulein (Sigma-Aldrich) dissolved in PBS on two consecutive days at a dose of 50 μg/kg. The pancreas from wild-type mice were harvested at two days after the last injection; while the pancreas from three *KRAS*$^{G12D/+}$ mice were harvested at four days and from three *KRAS*$^{G12D/+}$ mice were harvested at 21 days, respectively, after the last injection. Normal acinar cells were imaged from the pancreatic tissue of three C57BL/6 mice at 6-7 weeks. After the pancreatic tissue was harvest, it was fixed overnight in 10% neutral buffered formalin and embedded in paraffin.

STORM setup

STORM images were acquired using our custom-built system on the Olympus IX71 inverted microscope frame with a 100x, NA=1.4 oil immersion objective (UPLSAPO 100XO; Olympus) and the system has been described in detail previously [1, 2]. For single-color dSTORM imaging, the excitation laser at 642 nm (VFL-P-1000-642-OEM3; MPB Communications, Point-Claire, Quebec, Canada) was used at power density of ~2.5kW·cm^{-2} for STORM imaging. The exposure time was 20 milliseconds and a total frame number of 40,000 were used. During the image acquisition, a small amount of activation power (~1 μW) for 405 nm laser (DL405-050, CrystaLaser, Reno, NV) was added at 3001st frame and the power of 405 nm laser was gradually increased at a rate of 0.2% per 1000 frames. Two-color dSTORM imaging was conducted sequentially, where first 30,000 frames were acquired on Alexa Fluor 647 with an exposure time of 20 msec for each frame, followed by 30,000 frames of Cy3B with the same exposure time. The excitation laser of 561 nm at laser power of 0.8kW·cm^{-2} (VFL-P-200-560-OEM1, MPB Communications, Point-Claire, Quebec, Canada) was used for imaging Cy3B-labeled targets. Drift correction was independently performed every 200 frames (or 4 seconds) with fluorescent beads (Thermo Fisher Scientific, F8803) excited with 488 nm laser (DL488-150, CrystaLaser, Reno, NV) as fiduciary markers throughout each image acquisition process, based on our established method [2]. The imaging conditions (exposure time, power density, activation, frame number) remain the same for all experiments reported in this study.

STORM imaging buffer

STORM imaging buffer for cultured cells contains 10% (w/v) glucose (Sigma-Aldrich), 0.56 mg/mL glucose oxidase (Sigma-Aldrich), 0.17 mg/mL catalase (Sigma-Aldrich), 0.14M 2-mercaptoethanol (βME, Sigma-Aldrich). For FFPE tissue sections, to reduce the high background caused by the strong scattering of pathological tissue and match the refractive index, optical clearing process were conducted

before imaging by immersing the sample in 60% (v/v) 2,2'-thiodiethanol (TDE) for 20-30 minutes to make the sample transparent. For the STORM imaging buffer of FFPE tissue section, 60 % (v/v) TDE solution was used instead of water to match the tissue's index, and contains 10% (w/v) glucose (Sigma-Aldrich), 0.56 mg/mL glucose oxidase (Sigma-Aldrich), 0.17 mg/mL catalase (Sigma-Aldrich), 0.14M 2-mercaptoethanol (βME, Sigma-Aldrich), and 0.2 mM Cyclooctatetraene (COT, Sigma-Aldrich). The STORM imaging buffer for ultrathin tissue section contains the same reagents at the same concentration, but without TDE. The imaging buffer for TOTO-3 labeled DNA in tissue contains the same reagents at the same concentration, but without COT and βME. The imaging buffer was added into the sample dish right before imaging.

Western blotting

Western blot analysis was performed following the standard protocols with tubulin as loading control. In brief, cell lysates were resolved in SDS loading buffer and subjected to electrophoresis in SDS-polyacrylamide gels and transferred to the polyvinylidene difluoride (PVDF) membrane. After blocking with 5% non-fat milk for 1 hour, membranes were incubated with corresponding primary antibody (H3K9me3, abcam, 1:400; tubulin, Cell Signaling Technology, 1:3000; RNAP II, abcam, 1:1000) at 4°C overnight. The membranes were washed three times in PBS and incubated with horseradish peroxidase (HRP)-conjugated secondary antibody (abcam, 1:2000) for 1 hour at room temperature. Membranes were washed 3 times with 0.1% PBS before exposure. Detection was done using BIORAD Universal Hood II machine with ImageLab software (see Source Data for original scans).

Metaphase spreading

NIH3T3 cells reaching 70-80% confluency were treated with 0.1 μg/ml Colcemid (Thermo Fisher Scientific) for 1 hour, then trypsinized and washed with PBS. After centrifugation, cells were resuspended with 75 mM KCl for 20 minutes at 37°C. Cells were fixed with freshly made Carnoy's Fixative (3+1 v/v methanol/glacial acetic acid) three times. Cells suspension was dropped on an ice-cold clean slide and air-dried for one day. Slides were stained with KaryoMAX™ Giemsa Stain Solution (Thermo Fisher Scientific) and observed under the bright-field microscope.

Sample Preparation for 3D-SIM imaging

Three C57BL/6J wild-type mouse and three age-matched *Apc*$^{Min/+}$ mice were sacrificed and their small intestine tissue were removed, fixed in 2% paraformaldehyde solution in PBS and placed in 30% sucrose for cryoprotection. The tissue was then flash frozen in liquid nitrogen and then stored at -80°C. Prior to imaging, the frozen tissue was sectioned at 15 μm using a cryostat and stained with 4′,6-diamidino-2-phenylindole (DAPI) and mounted in Gelvatol. A 3D stack of fluorescence images was acquired using N-SIM (Nikon) with a scanning depth of 18 μm at a step size of 0.12 μm. The 3D-SIM images were reconstructed using the image reconstruction software on the N-SIM system.

Ultrathin tissue section

A C57BL/6J wild-type mouse was sacrificed and the small intestine tissue was removed, washed with PBS. Two adjacent equal segments were taken. One piece was fixed in 10% formalin for 2 hours and then processed using standard paraffin-embedding protocol and sectioned at 3 μm. Immunofluorescence staining and PathSTORM imaging were performed on the FFPE tissue section as described in the Experimental Procedures of the main text. The second piece was fixed in 2% paraformaldehyde for 2 hours, then in 30% sucrose for 24 hours, and stored in liquid nitrogen. Then an ultrathin section (~700 nm) was cut using an Ultramicrotome (Reichert Ultracut). The standard immunofluorescence staining was performed and STORM imaging was done in the same way as previously described in cultured cells [1].

DNA and immunofluorescence staining for cultured cells

DNA staining in cell cultures was performed by using the Click-iT Plus EdU (5-ethynyl-2′-deoxyuridine) Alexa Fluor Imaging Kit (Thermo Fisher Scientific), as described in detail previously [1, 3]. In brief, cells were incubated with 1 μM EdU contained medium for 24 hours, after fixation and permeabilization, cells were blocked with 3% BSA and incubated with EdU Click-iT Plus reaction cocktail for 30 minutes following the manufacturer's instruction. DNA were detected by Azide CF-568. For two-color staining of DNA and proteins, after being washed out of the reaction cocktail, cells were incubated with the primary antibody at 4 °C overnight. Cells were then washed 3 times with the washing buffer for 5 minutes per wash, and the corresponding Alexa-647 conjugated secondary antibodies were added to the sample in blocking buffer and incubated for 2 hr at room temperature. Cells were washed again 3 times with washing buffer and once with PBS for 5 min per wash and stored in PBS before imaging.

The performance of TOTO-3 for STORM imaging was benchmarked against Alexa 647 by labeling DNA in cultured NIH3T3 cells. DNA was stained with Alexa 647 as described above. Cells were fixed with 4% PFA and permeabilized with 0.2% Triton-X100. Cells stained with TOTO-3 were treated with 50 μg/mL DNase-free RNase at 37°C for 30 minutes to remove the RNAs in the cells. After 3 washes, cells were incubated with 100 nM TOTO-3 for 30 minutes at room temperature, then washed 3 times with PBS and ready for STORM imaging. The recipe of the imaging buffer for TOTO-3 in cells was the same as in tissue sections described above.

DNA staining at different stages of cell cycle

Cells at ~ 60% confluency were incubated with 1 μM EdU for 24 hours, then the medium was changed to the fresh medium containing 10 μM BrdU (Thermo Fisher Scientific) and the cells were incubated for another 1 hour. Cells were fixed with 4% PFA for 15 minutes and permeabilized with 0.2% Triton X-100 for 10 minutes. DNA was denatured by incubating with 2N HCl for 30 minutes at 37°C to make the nucleotides accessible for the antibody, then neutralized by incubating with Tris-HCl buffer pH 7.5 for 20 minutes. The cells were blocked in 3% BSA for 2 hours and incubated with BrdU antibody (Cell

Signaling Technology) at 4°C overnight. After washing, cells were incubated with Cy3B-conjugated secondary antibody (1:200 Cy3B reactive dye, GE Healthcare, PA63101; Donkey anti-mouse antibody, Jackson ImmunoResearch, 715-005-151) for 2 hours at room temperature. Cells were washed 3 times, and EdU was detected by Click-iT Plus EdU Alexa Fluor Imaging Kit as descried above. Azide Alexa Fluor 647 was then used to detect EdU labeled DNA.

RNA interference (RNAi)

NIH3T3 cells were cultured in DMEM with 10% FBS at 37 °C and 5% CO_2. Before transfection, cells were plated on 2 cm MatTek dish until they reach 70% confluency. Cells were transfected with 50 nM Suv39h1 siRNA (Integrated DNA Technologies) using RNAimax transfection reagent (Thermo Fisher Scientific) diluted in OptiMEM (Gibco), after 24 hours, medium was exchanged to fresh medium with 1 μM EdU and incubated for another 24 hours. Cells were fixed with 4% PFA for 15 minutes and permeabilized with 0.2% Triton X-100 for 10 minutes. Cells were stored in PBS for DNA or immunofluorescence staining.

DRB or serum treatment

Cells were treated with 20 μM DRB (5,6-dichloro-1-β-D ribofuranosylbenzimidazole, Sigma-Aldrich) diluted in full culture medium or 20% FBS in DMEM at 37 °C and 5% CO_2 for 12 hours. After treatment, cells were fixed with 4% PFA for 15 minutes and permeabilized with 0.2% Triton X-100 for 10 minutes. Ser5 phosphorylated RNAPII CTD (active form of RNAPII) was stained by immunofluorescence with Alexa647-conjugated secondary antibody for STORM imaging, as described above.

Hematoxylin & Eosin (H&E) Staining

FFPE sections were deparaffinized in xylene and rehydrated in graded ethanol/water followed by distilled water. Then the sections were stained with Harris hematoxylin (Anatech Ltd), washed by distilled water, then treated with 10% glacial acetic acid (Fisher Scientific) and Scott's Tap Water Substitute (1 min, Cancer diagnostics Inc). Then the tissue was stained with Eosin-Y (Anatech Ltd), followed by three washes with 70% ethanol, three washes with 100% ethanol and three washes with xylene. Finally, stained sections were cover slipped with microscope cover glasses (Fisherbrand, Fisher Scientific) and dried at room temperature.

Simulation of two-color co-localization for randomly distributed molecules

To simulate the randomly distributed clusters, we first calculated the number of localizations for the two-color channels (DNA and active RNAPII) and the area of the cell nucleus obtained from the experimental data of cells treated with SUV39h1. Assuming the same number of localizations and area of nucleus, we simulated randomly distributed molecules within the same area and calculated the cross-correlation map between the two-color image from both simulated and experimental data using function "normxcorr2" in MATLAB.

Supplementary Figures

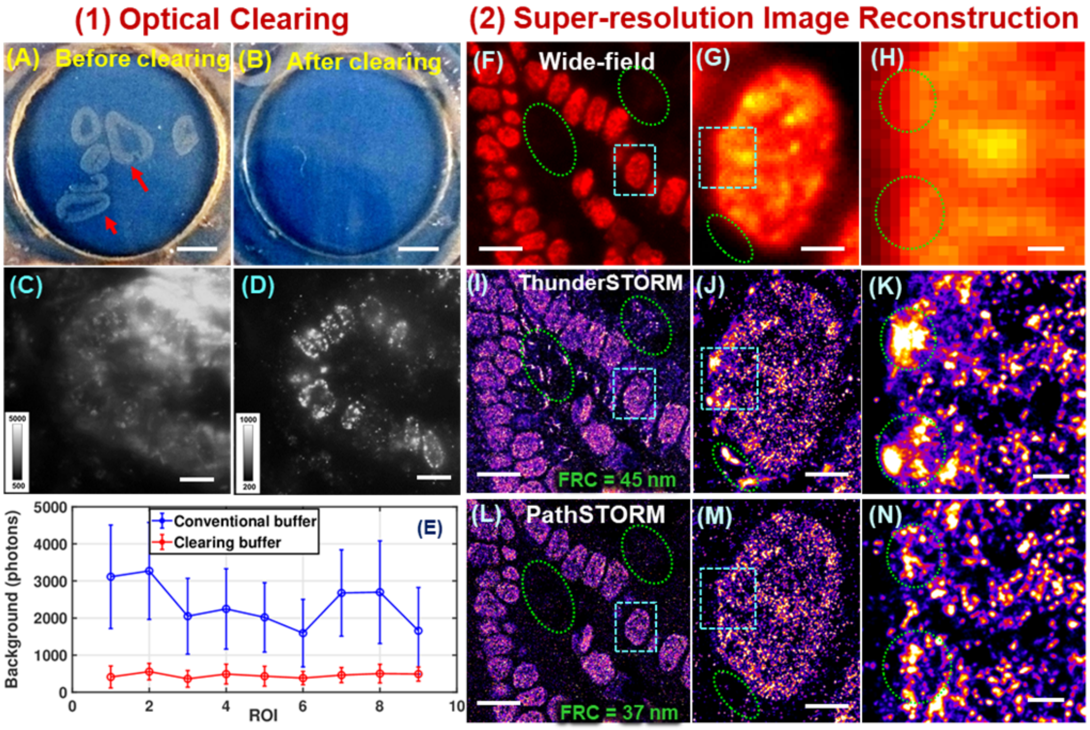

Supplementary Figure 1. Workflow of PathSTORM. **(1)** Optical clearing: **(A, C)** Mouse intestinal tissue section (3μm) before optical clearing and the raw image in the standard aqueous-based STORM imaging buffer at a power density of ~3kW/cm^2. **(B, D)** After optical clearing, tissue appears transparent and the raw image shows dramatically reduced background under index-matched imaging buffer. **(E)** Comparison of average background signals (open circle) before and after clearing from 9 different regions of interest (ROIs). Error bar: Standard deviation of background signal for each ROI. There is about 5-fold reduction in background after clearing. **(2)** Super-resolution image (H4Ac) reconstruction that corrects for heterogeneous background and decomposes overlapping emitters. **(F-H)** Conventional wide-field image andthe reconstructed super-resolution images with **(I-K)** a conventional method (ThunderSTORM) and **(L-N)** PathSTORM. **(G-H)**, **(J-K)** and **(M-N)** are the progressively zoomed images of **(F, I, L)** in the blue square, respectively. The scale bars in (A-B), (C-D, F, I, L), (G, J, M) and (H, K, N) represent 2 mm, 10 μm, 2μm and 500 nm, respectively. The Fourier Ring Resolution (FRC) resolution is shown at the bottom of (I, L). Comparison of the same areas in the conventional diffraction-limited wide-field images and the reconstructed STORM images by ThunderSTORM and PathSTORM is shown in (F-N). The image reconstructed by conventional method (implemented by ThunderSTORM) (I-K) shows apparent image artifacts that are not present in the diffraction-limited image (F-H). In the zoomed super-resolution image (J-K), the artifacts (circled in green) distort the image of the actual chromatin structure. In contrast, the image reconstructed by PathSTORM (L-N) shows good agreement with the diffraction-limited image (F-H) with a higher image resolution. These results showed that PathSTORM increases the image resolution and reduces the image artifacts present in chromatin structure and background.

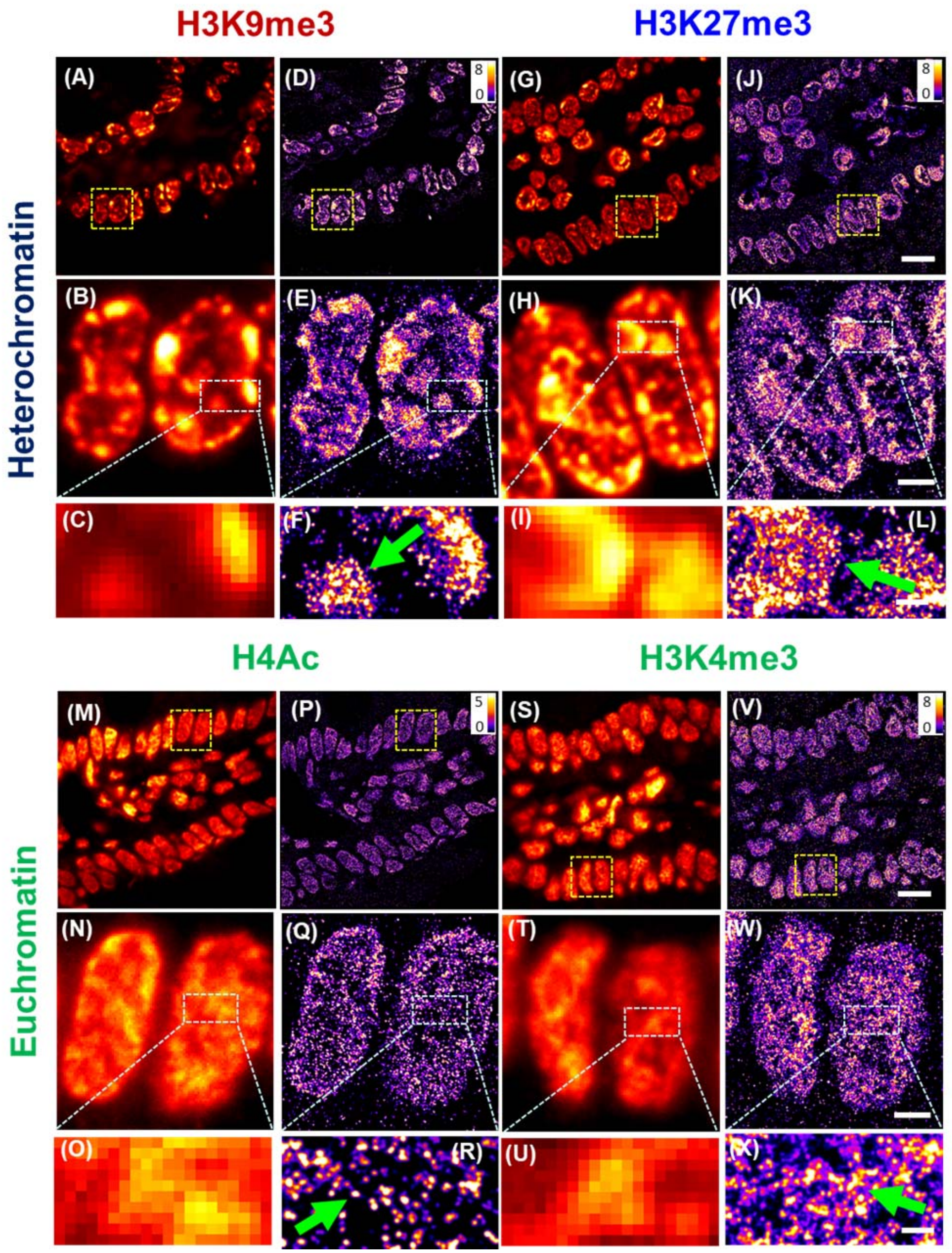

Supplementary Figure 2. Representative (**A**, **G**, **M**, **S**) conventional wide-field and (**D**, **J**, **P**, **V**) super-resolution images of (**A, D, G, J**) higher-order heterochromatin structures marked by transcriptionally repressive histone proteins (H3K27me3 and H3K9me3) and (**M, P, S, V**) higher-order euchromatin structures marked by transcriptionally active histone marks (H4Ac and H3K4me3) on the pathological tissue of mouse intestine. (**B, E, H, K, N, Q, T, W**) and (**C, F, I, L, O, R, U, X**) are the progressively zoomed images. The scale bars of the original and magnified images represent 10 µm, 2 µm and 500 nm, respectively. The green arrows indicate the characteristic condensed large nanoclusters formed by heterochromatin and more uniform or spatially diffuse nanoclusters formed by euchromatin.

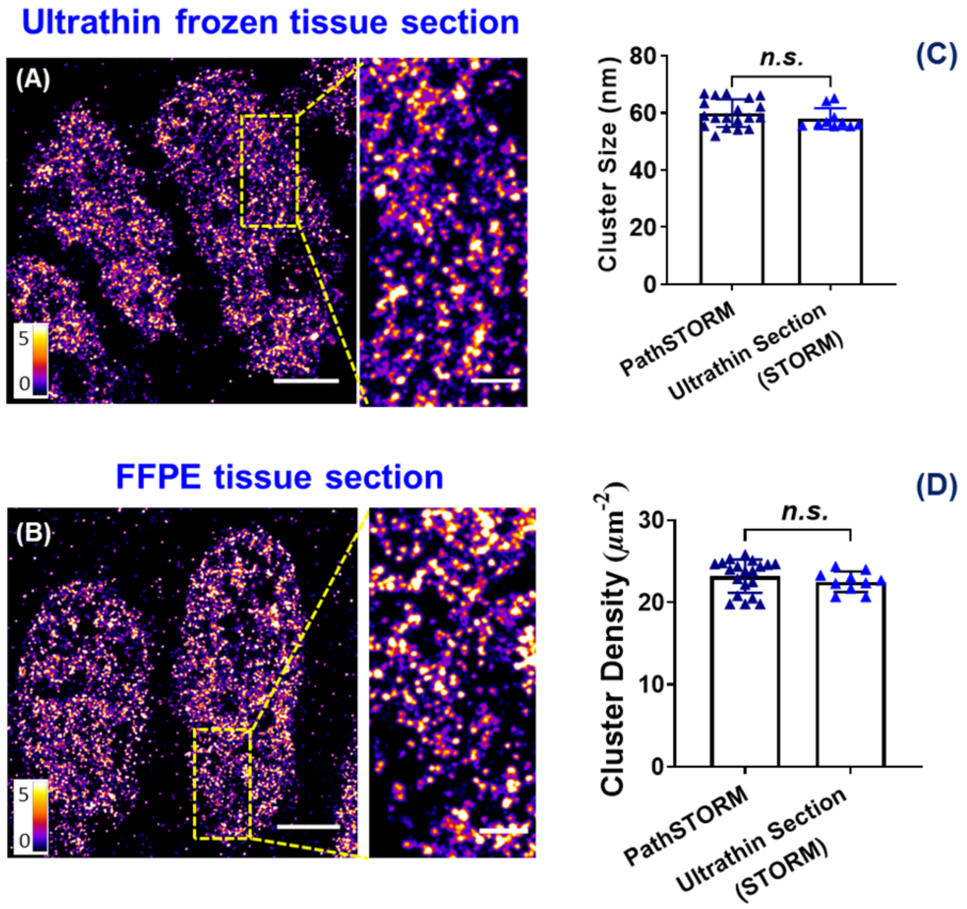

Supplementary Figure 3. (A-B) Comparison of reconstructed STORM images of euchromatin structure (stained with euchromatin marker H3K4me3) from ultrathin frozen section (**A**) and FFPE section (**B**) of the same mouse small intestine stained with H3K4me3. The scale bars in original and magnified images are 2 µm and 500 nm, respectively. (**C-D**) Comparison of euchromatin cluster size (C) and cluster density (D) for the tissue from two closely adjacent areas with one processed as FFPE tissue section and imaged with PathSTORM (n = 20 cells); another processed as ultrathin frozen tissue section and imaged with standard STORM imaging (n = 10 cells). The ultrathin frozen section (700 nm) was cut with ultra-microtome, stained and imaged with standard protocols used for cultured cells. The FFPE tissue section was cut at 3µm with a microtome, stained, imaged and reconstructed with PathSTORM method described in the main text. Error bars: mean ± 95% CI. *P* values were determined using Mann-Whitney test.

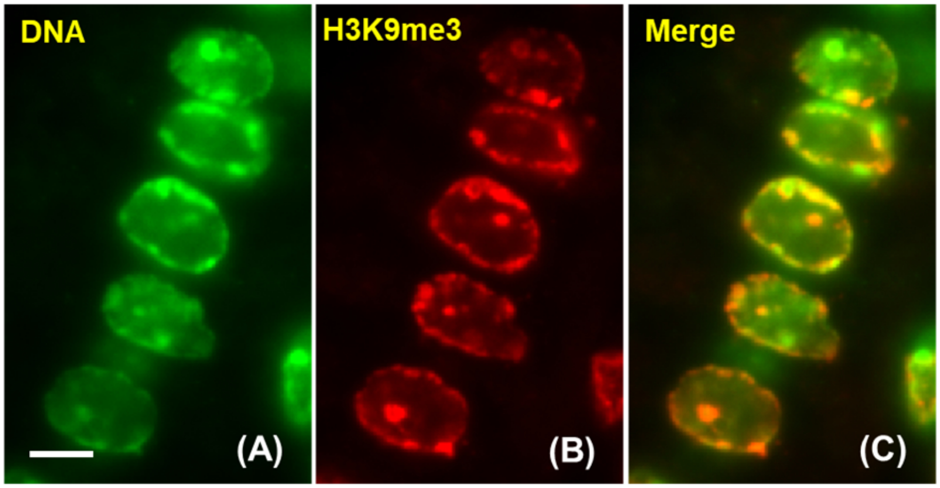

Supplementary Figure 4. Conventional wide-field fluorescence images of (**A**) DNA (stained with DAPI) and (**B**) H3K9me3 (Cy3B) and (**C**) the merged image on the intestinal epithelial tissue from a wild-type mouse. Scale bar: 5 µm. The histone mark H3K9me3 largely overlaps with the condensed regions of the DNA in the cell nuclei *in vivo*.

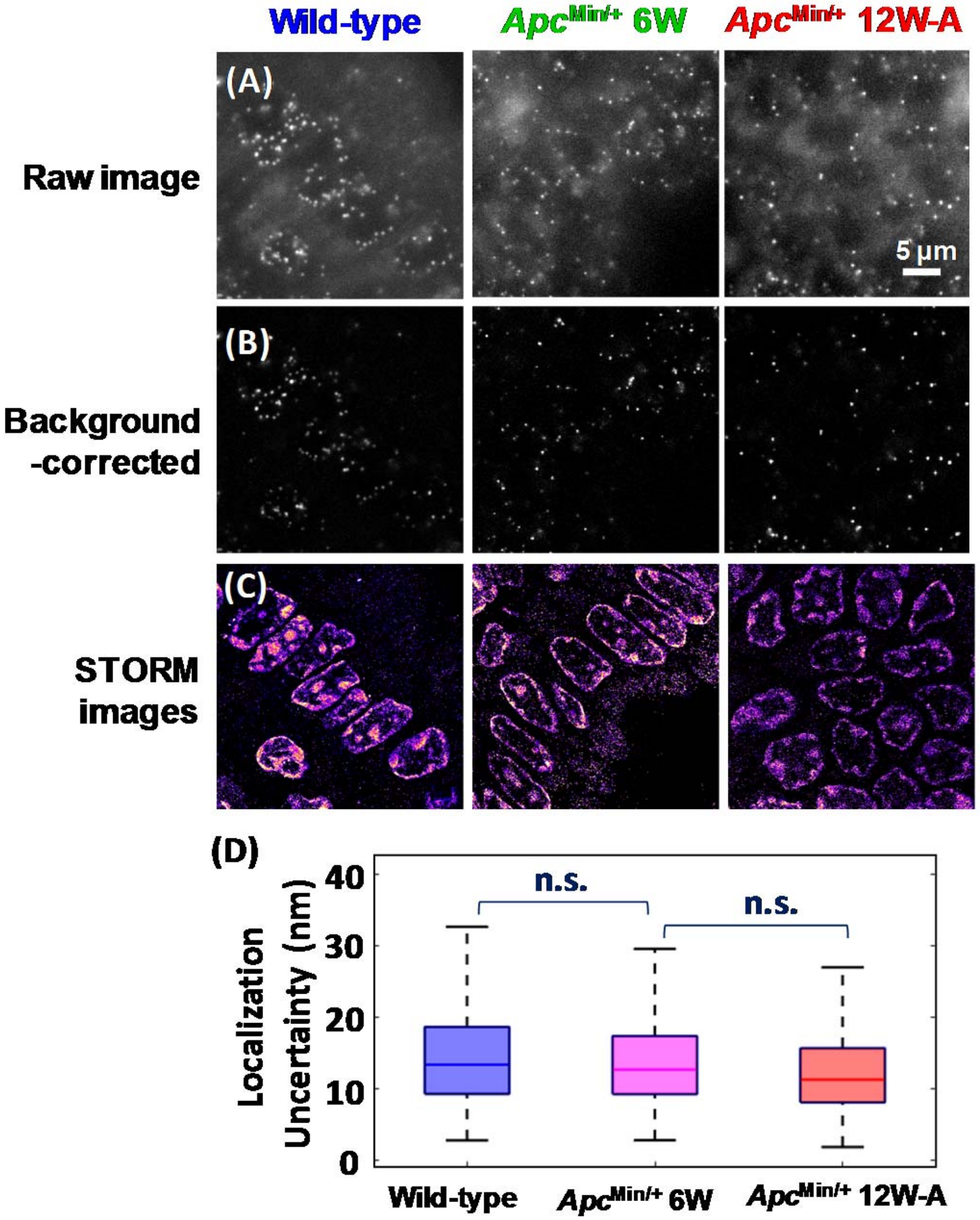

Supplementary Figure 5. Imaging performance of PathSTORM on H3K9me3-dependent heterochromatin in the wild-type and *Apc*$^{Min/+}$ mouse intestinal tissue. (**A-C**) Single-frame raw images after optical clearing under optimized PathSTORM imaging conditions (A) before and (B) after background correction, and (C) the reconstructed STORM images of normal-appearing cells from wild-type and 6-week *Apc*$^{Min/+}$ mice and tumor cells (adenoma) from 12-week *Apc*$^{Min/+}$ mice. (**D**) Box-and-whisker plot of the localization uncertainty defined as the standard deviation of the localization precision for the nuclei analyzed in each group. In the box plot, the central line of the box indicates the median; the bottom/top edge of the box indicate 25th/75th percentiles. The number of localized spots was used to generate the box plot for each group (2.24×10^7, 1.16×10^7 and 1.10×10^7, respectively). There was no statistical significance between any of the two groups. All *P* values were determined using Mann-Whitney test.

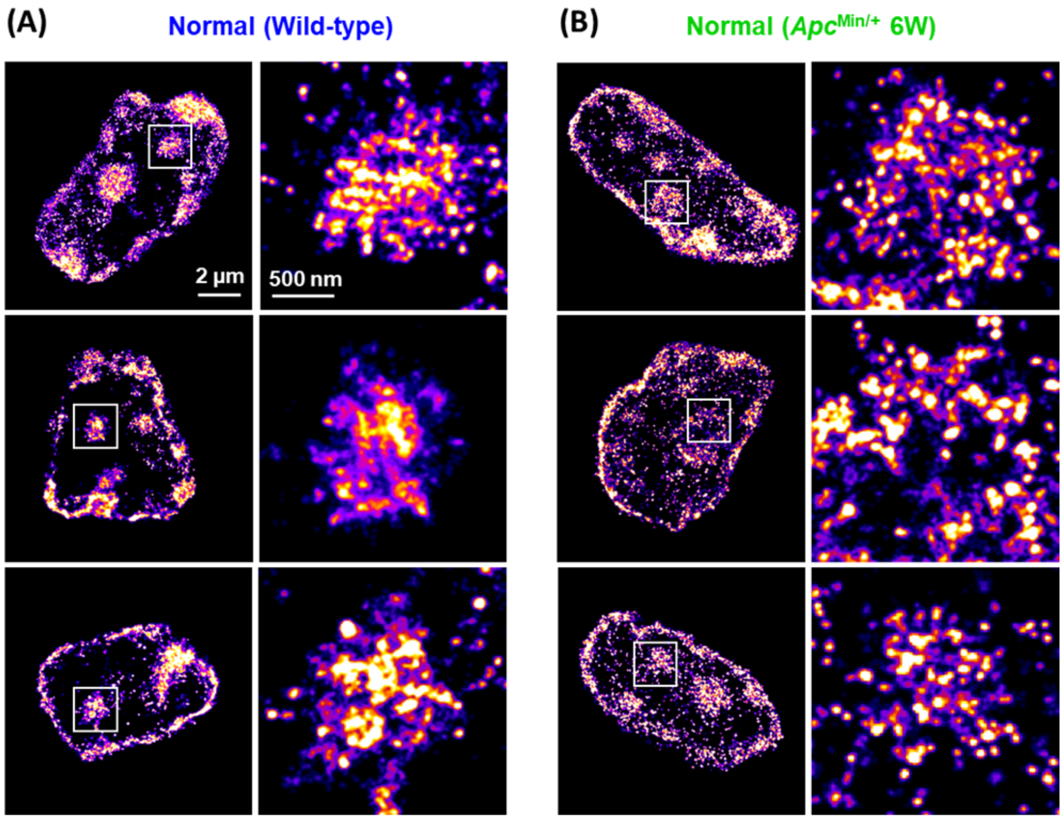

Supplementary Figure 6. Representative STORM images of heterochromatin structure (marked by H3K9me3) of **(A)** normal intestinal epithelial cells from wild-type mice and **(B)** normal-appearing intestinal epithelial cells from *Apc*$^{Min/+}$ mice at 6 weeks containing similar number of localizations per nucleus by adjusting the number of image frames accumulated in the final reconstructed image[4]. The heterochromatin structure from 6-week *Apc*$^{Min/+}$ mice appears to be more spatially segregated and diffused, compared to that from wild-type mice.

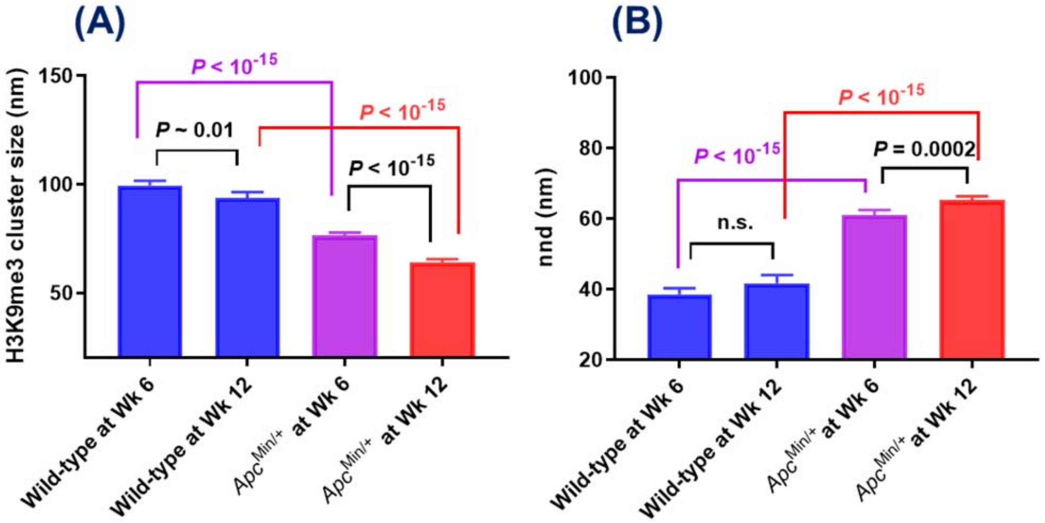

Supplementary Figure 7. The comparison of (A) H3K9me3 cluster size and (B) nearest neighbor distance (nnd) from intestinal epithelial cell nuclei of normal tissue from wild-type mice at 6 weeks and 12 weeks, compared to the age-matched *Apc*$^{\mathrm{Min/+}}$ mice. The cluster size of H3K9me3 only shows marginal difference, and nnd shows no difference between 6-week (n = 204 normal epithelial nuclei analyzed) and 12-week (n = 241 normal epithelial nuclei) from wild-type mice. The age-related difference is significantly smaller compared to carcinogenesis-associated difference. Error bars: mean ± 95% CI. P values were determined using Mann-Whitney test.

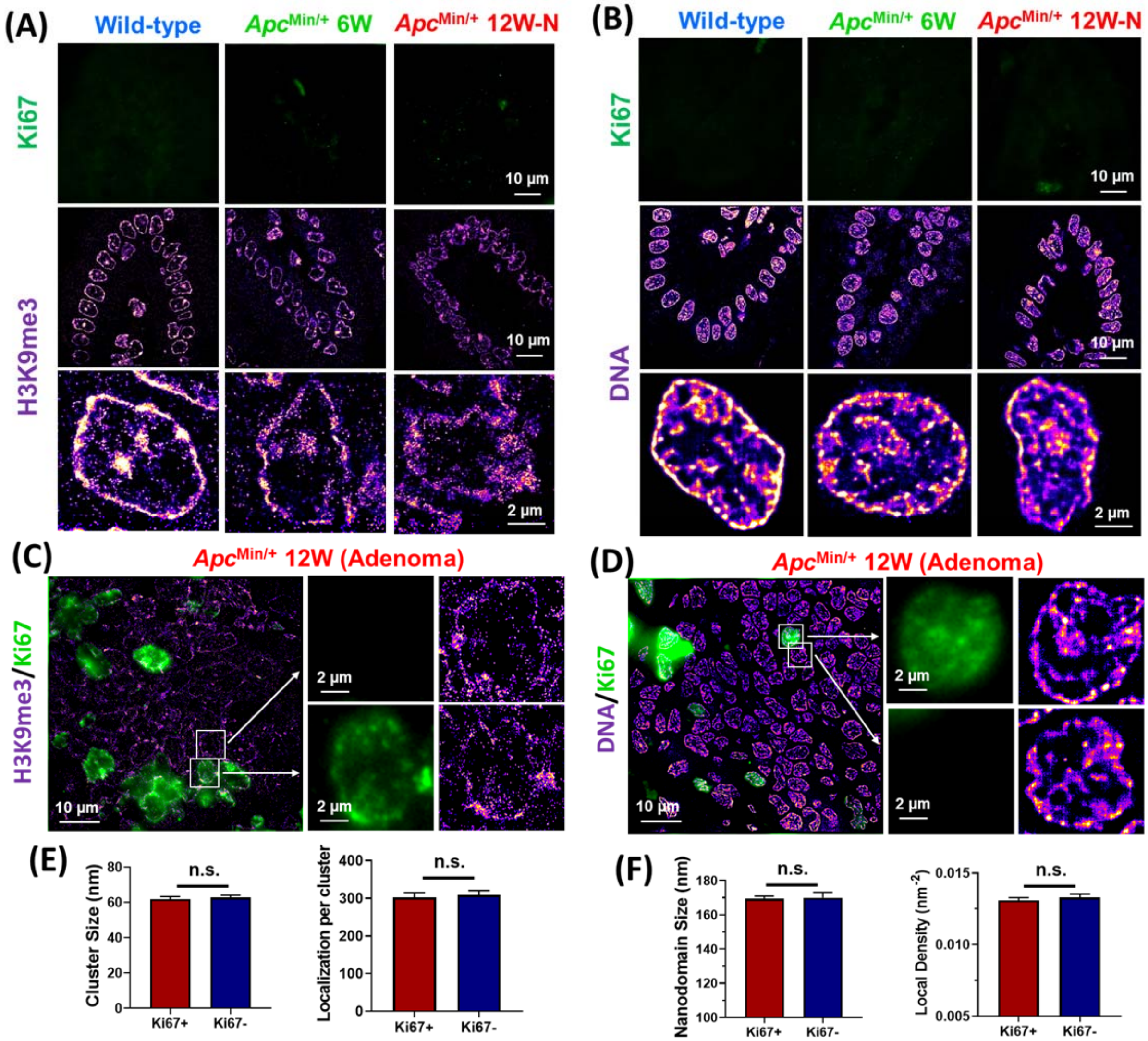

Supplementary Figure 8. Super-resolution imaging of H3K9me3 and DNA associated with cellular proliferation. **(A-B)** Chromatin structure labeled by (**A**) H3K9me3 and (**B**) DNA overlaid with Ki67 staining (green) in normal-appearing intestinal epithelial cells from wild-type, 6-week *Apc*$^{Min/+}$, and 12-week *Apc*$^{Min/+}$ mice. All of the cells analyzed were normal-appearing cells non-proliferating intestinal epithelial cells in the villi regions with negative Ki67 staining. **(C-D)** Chromatin structure labeled by (**C**) H3K9me3 and (**D**) DNA overlaid with Ki67 staining in adenoma lesions in 12-week from *Apc*$^{Min/+}$ mice. The cellular proliferation status was labeled with Ki67 marked in green color. **(E)** Statistical analysis of H3K9me3 cluster size and number of localizations per cluster between cells with negative and positive Ki67 signals in tumor cells (adenoma) from 12-week from *Apc*$^{Min/+}$ mice. No statistical difference was observed Ki67- (n = 170 cells) and Ki67+ (n = 133 cells) tumor cells. (**F**) DNA nanodomain size and local density of DNA (Voronoi polygon density) between tumor cells (adenoma) with negative and positive Ki67 signals from 12-week *Apc*$^{Min/+}$ mice. No statistical difference was observed between Ki67- (n = 76 cells) and Ki67+ (n = 140 cells) tumor cells. Error bars: mean ± 95% CI. P values were determined using Mann-Whitney test.

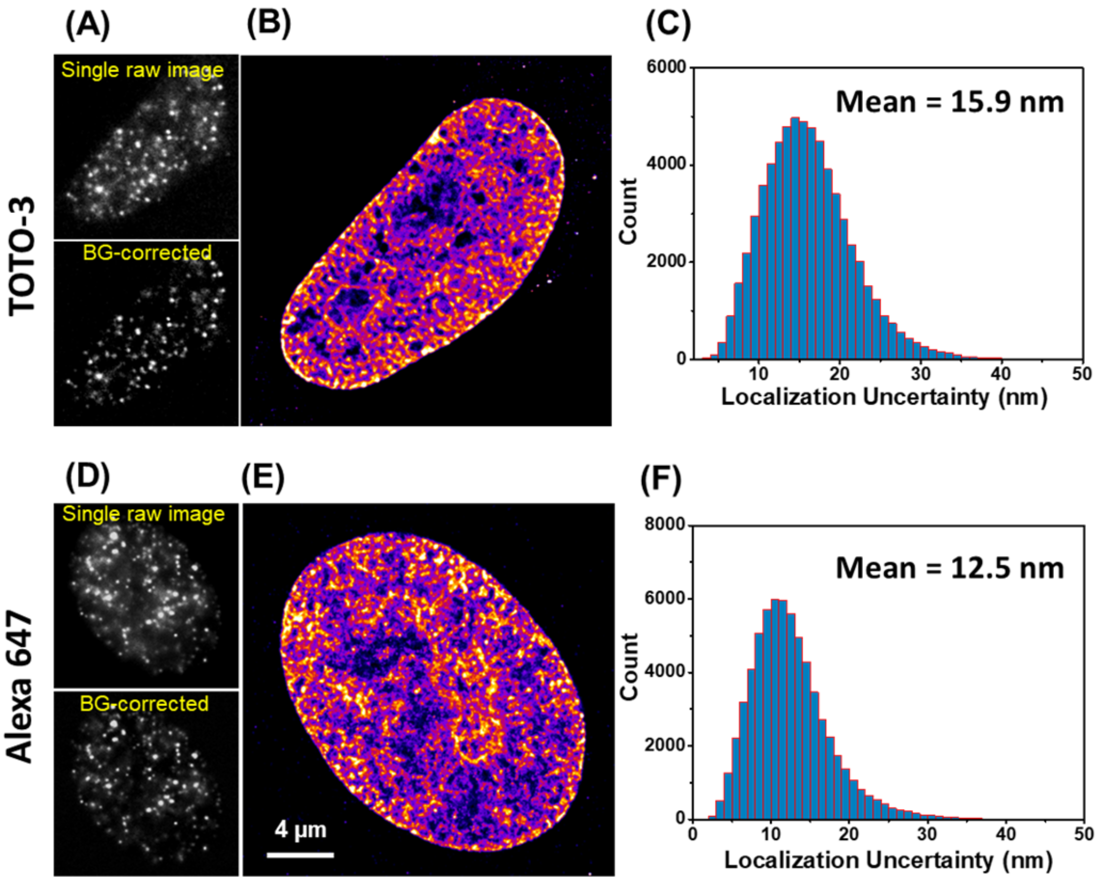

Supplementary Figure 9. Comparison of photo-switching performance of TOTO-3 and Alexa 647-labeled DNA in cultured NIH-3T3 cells. (**A**) Single-frame raw image of TOTO-3 in modified STORM imaging buffer before and after background correction. (**B**) Reconstructed STORM image of TOTO-3 labeled DNA. **(C)** Histogram of localization precision (mean value = 15.9 nm) corresponding to an optical resolution of ~37 nm. **(D)** Single-frame raw image of Alexa647-labeld DNA under standard STORM imaging condition before and after background correction. (**E**) Reconstructed STORM image of Alexa647-labeled DNA by Click-iT Plus EdU imaging Kit. (**F**) Histogram of localization precision (mean value = 12.5 nm) corresponding to an optical resolution of ~29 nm. Overall, TOTO-3 shows satisfactory photo-switching performance for STORM imaging in the appropriate buffer, with a slight reduction in the image resolution compared to that of Alexa 647 (37 nm vs. 29 nm in our test).

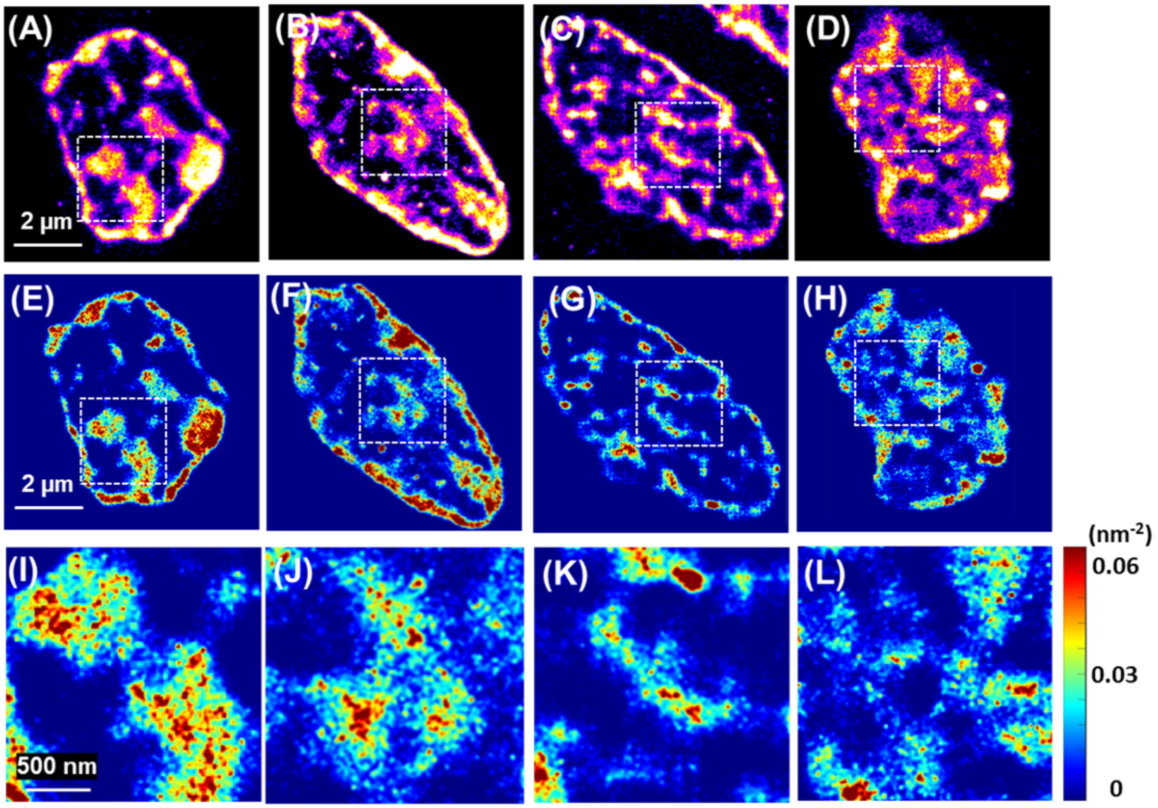

Supplementary Figure 10. Voronoi tessellation analysis. (**A-D**) STORM images of DNA folding from normal cells from wild-type, histologically normal-appearing cells from 6-week and 12-week *Apc*$^{Min/+}$, and tumor cells from 12-week *Apc*$^{Min/+}$ mice. (**E-H**, **I-L**) Local density map (Voronoi polygon density) of the original and magnified STORM images.

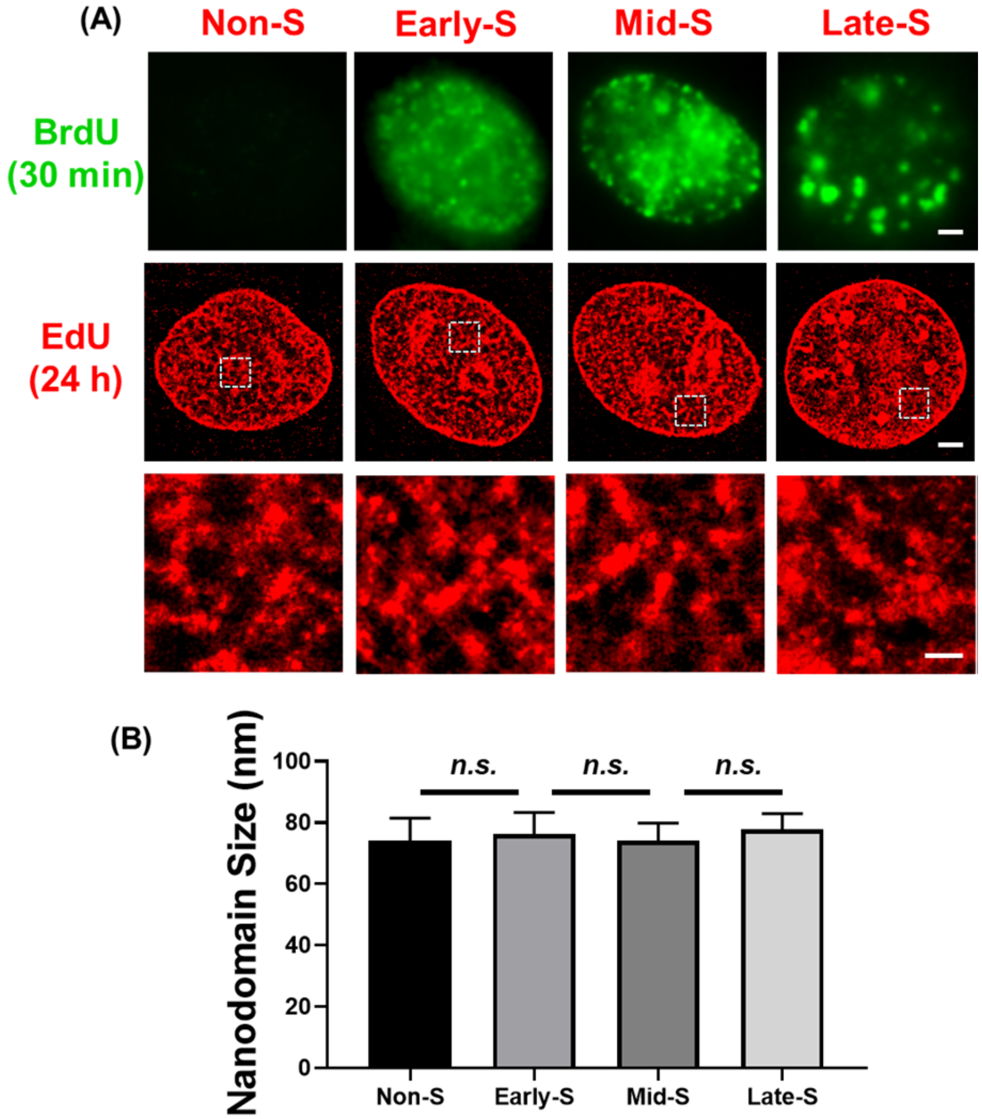

Supplementary Figure 11. (A) Super-resolution images of DNA structure at different cell cycles. BrdU is used to mark cells at S-phase; EdU is used to stain genome-wide DNA. The scale bars represent 2 µm, 2 µm, and 500 nm, respectively. **(B)** Statistical analysis of DNA nanodomain size of different cell cycles (n = 12, 11, 13, 11 cells, respectively). There was no statistical significance in the average size of the nanodomains. Error bars: mean ± 95% CI. *P* values were determined using Mann-Whitney test.

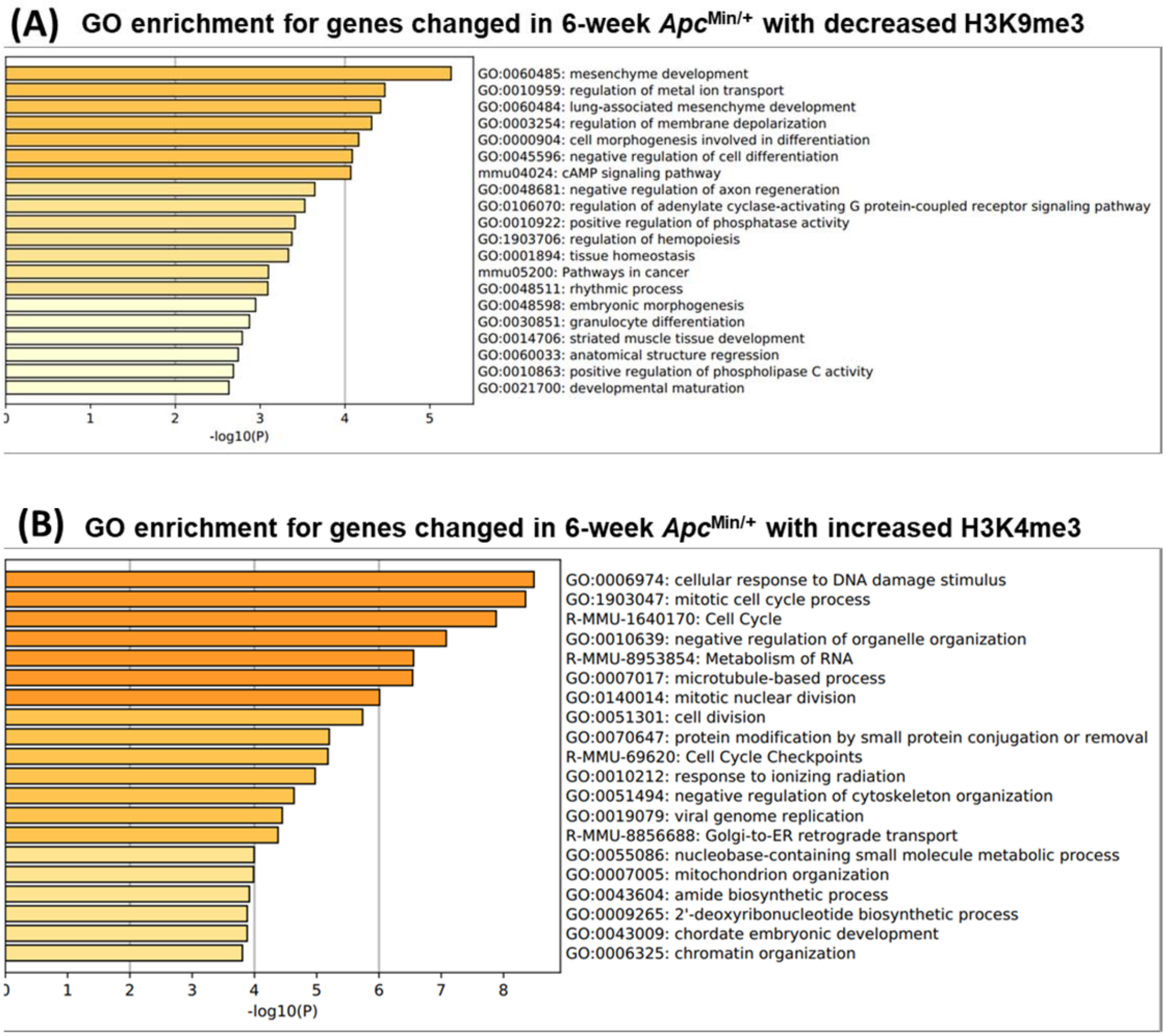

Supplementary Figure 12. (A) Gene ontology (GO) analysis for overlapping up-regulated genes with reduced occupancy of H3K9me3 in 6-week *Apc*$^{Min/+}$ mice. **(B)** GO analysis for overlapping up-regulated genes with increased occupancy of H3K4me3 in 6-week *Apc*$^{Min/+}$ mice. GO analyses were performed using Metascape software[5].

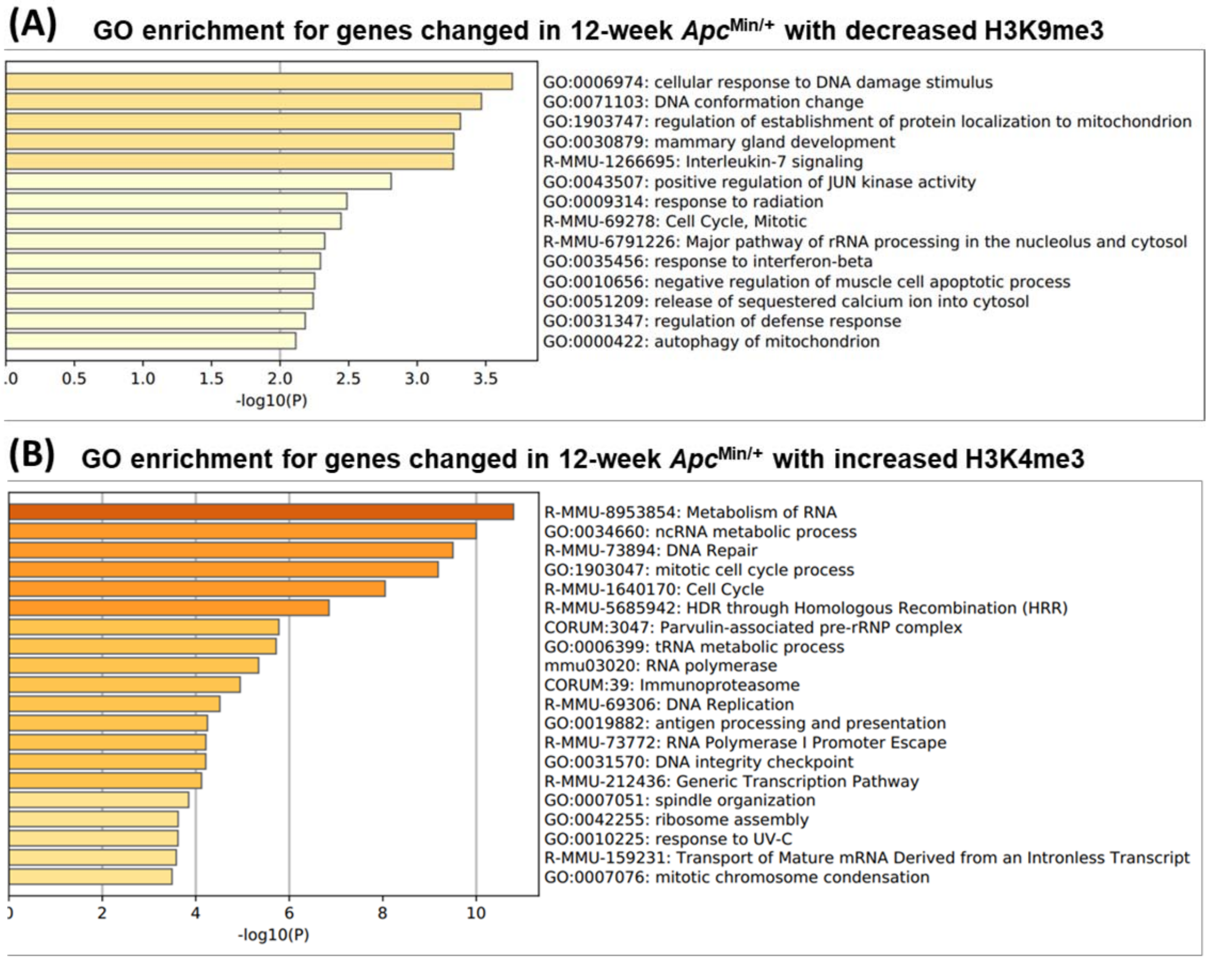

Supplementary Figure 13. (A) Gene ontology (GO) analysis for overlapping up-regulated genes with reduced occupancy of H3K9me3 in 12-week *Apc*$^{Min/+}$ mice. **(B)** GO analysis for overlapping up-regulated genes with increased occupancy of H3K4me3 in 12-week *Apc*$^{Min/+}$ mice. GO analyses were performed using Metascape software[5].

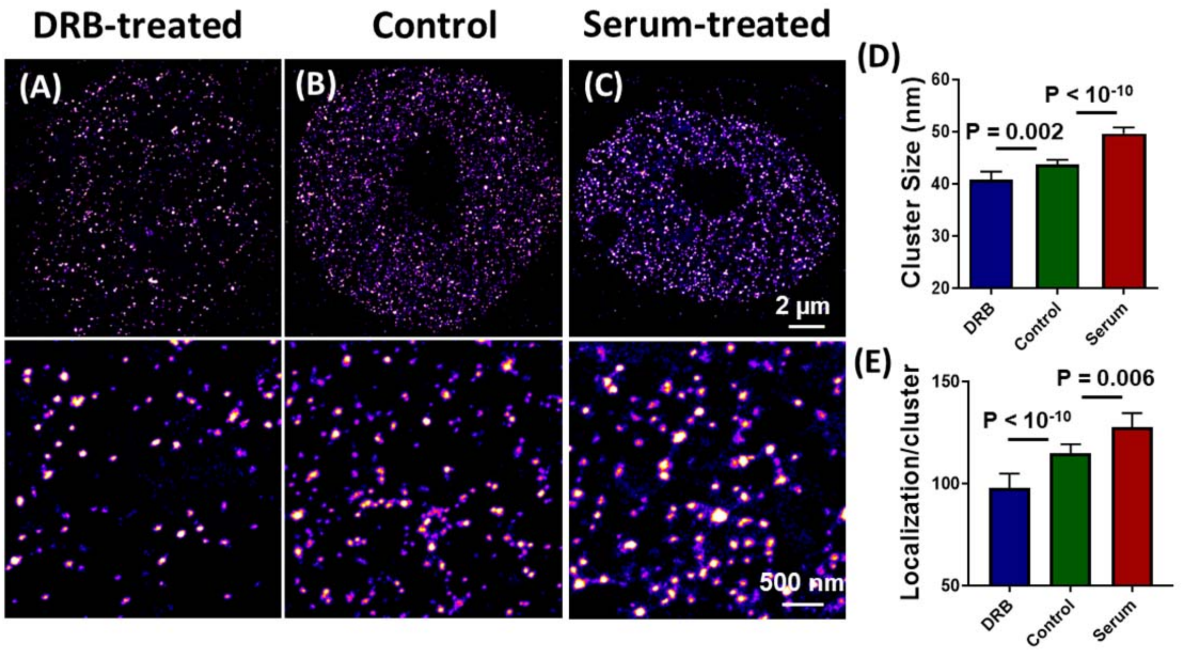

Supplementary Figure 14. Correlation between transcription activity and active RNAPII clusters. (**A-C**) STORM images of active RNAPII in cells treated with DRB (inhibiting transcription) or serum (stimulating transcription), respectively. (**D-E**) Statistical analysis of active RNAPII cluster size and number of localizations per cluster in control cells and (n = 31) cells treated with DRB (n = 23) or serum (n = 31). Increased active RNAPII cluster size and number of localizations per cluster correlated with increased transcription activities. Error bars, mean ± 95% CI. P values were determined using Mann-Whitney test.

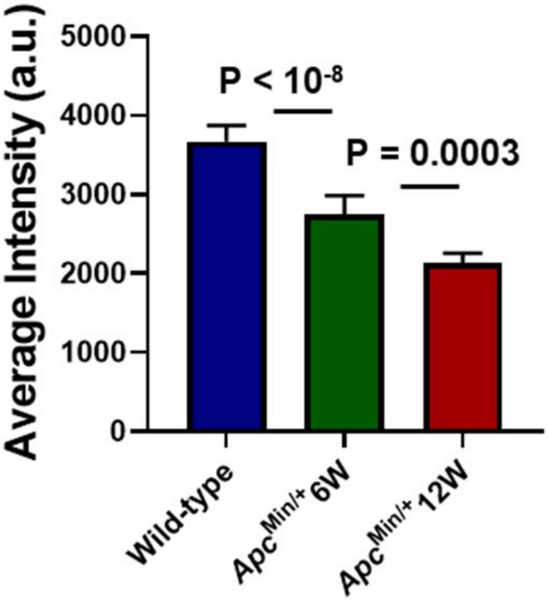

Supplementary Figure 15. Average fluorescent intensity of SUV39h1 from conventional wide-field images of normal-appearing intestinal epithelial cells from wild-type mice and 6-week *Apc*$^{Min/+}$ mice and tumor cells from 12-week *Apc*$^{Min/+}$ mice. The images were taken with the same laser power and exposure time. The intensity for each nucleus was calculated by averaging all the intensity values for the nucleus (n = 87, 95, 118 cells, respectively). Error bars: mean ± 95% CI. P values were determined using Mann-Whitney test.

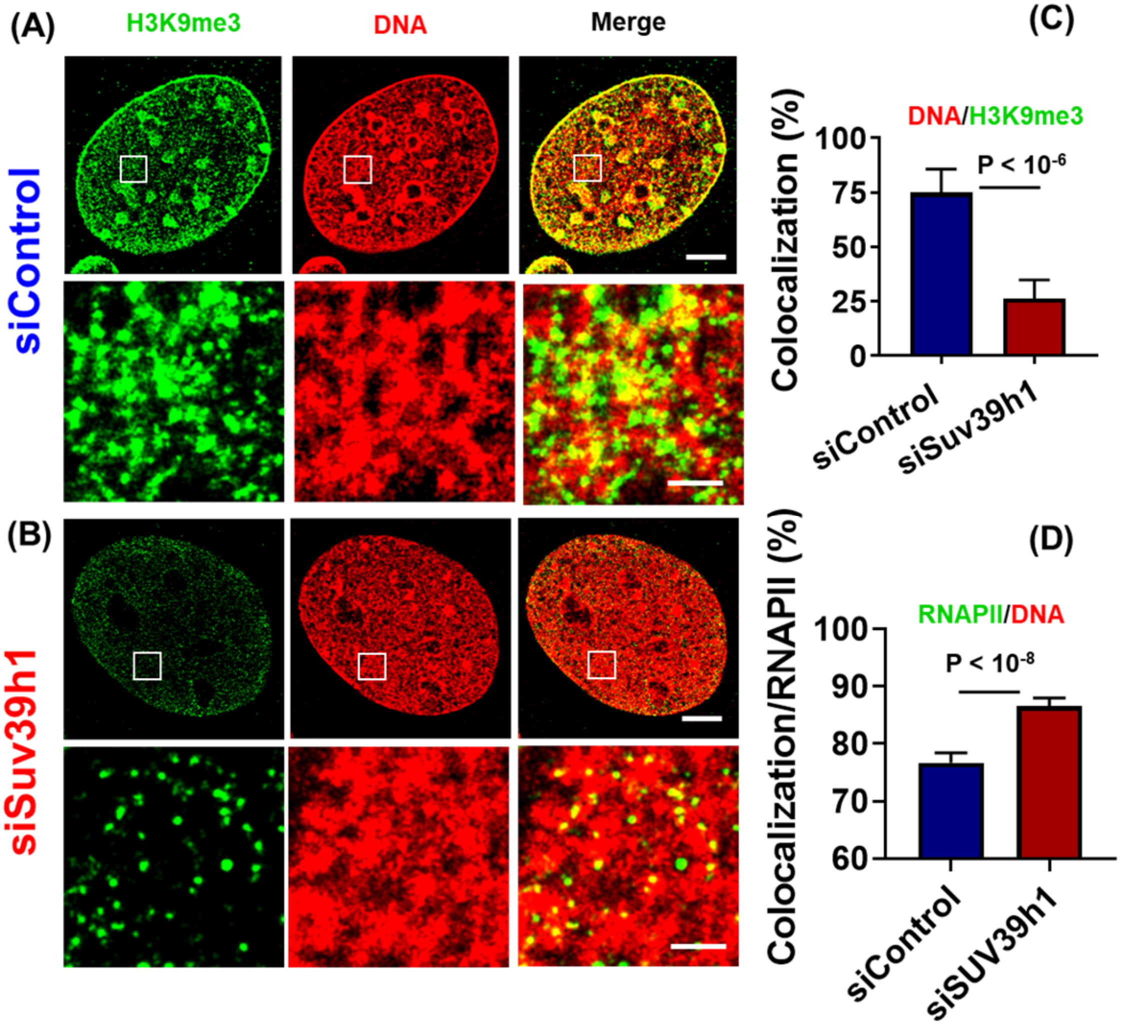

Supplementary Figure 16. (A-B) Representative two-color STORM images showing the spatial relationship between DNA (red channel) and H3K9me3 (green channel) in control and SUV39h1 knockdown cells. Scale bars: 2 µm, 500 nm in the original and magnified images, respectively. **(C)** Quantitative analysis of the percentage of DNA that overlaps with H3K9me3 in control (n = 12) and SUV39h1 knockdown (n = 13) cells. These data confirmed that after SUV39h1 knockdown, H3K9me3-dependent heterochromatin clusters became significantly smaller, with significantly less regions co-localized with DNA. **(D)** Co-localization analysis for RNAPII vs. DNA, or the percentage of active RNAPII that overlaps with DNA, in controls (n = 14) and cells with SUV39h1 knockdown (n = 27). Error bars: mean ± 95% CI. P values were determined using Mann-Whitney test.

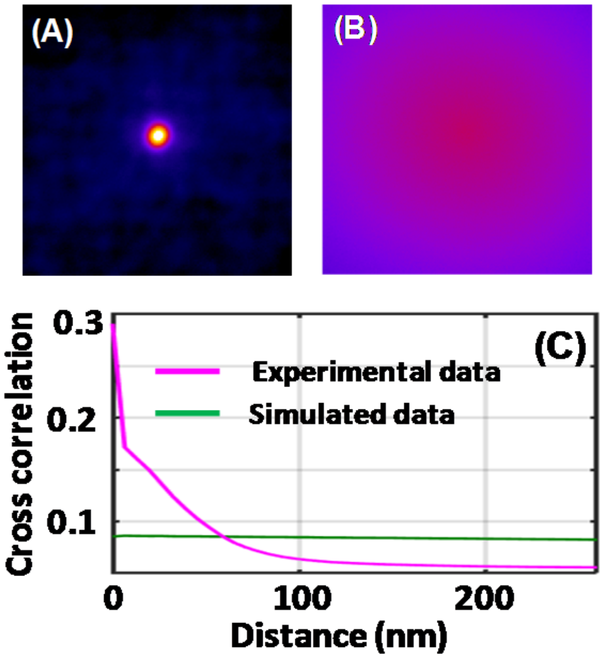

Supplementary Figure 17. (A-B) Cross-correlation maps of two-color STORM images from (**A**) the experimental data of NIH-3T3 cells treated with SUV39h1 and (**B**) the simulated data of randomly distributed molecules assuming the same number of localizations and the area of the nucleus for each of two-color channels as the experimental data. **(C)** Radial distribution from the cross-correlation map for the experimental data (magenta) and the simulated data with randomly distributed molecules (green). This data supports that the increased co-localization between DNA and RNAPII upon SUV39h1 knockdown is not by chance.

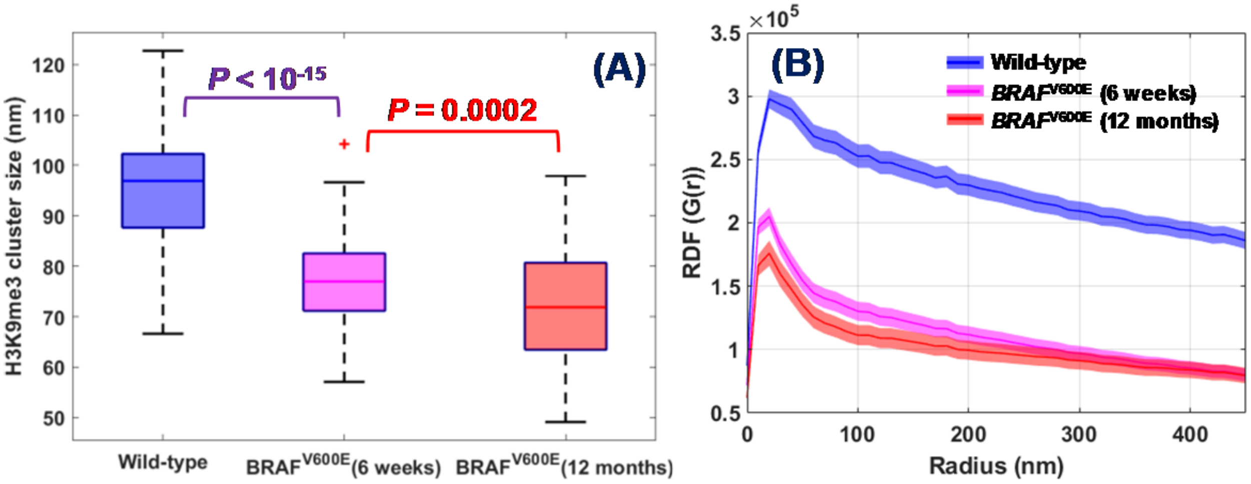

Supplementary Figure 18. (A) Box-and-whisker plots of the H3K9me3 cluster size from intestinal epithelial cell nuclei of normal tissue from wild-type mice (n = 154), histologically normal-appearing tissue from 6-week *BRAF*^{V600E} mice (n = 141) and tumor (adenoma) from 12-month *BRAF*^{V600E} mice (n = 100). In the box plot, the central line of the box indicates the median; the bottom/top edge of the box indicate 25th/75th percentiles. (**B**) Average radial distribution function (RDF) for all nuclei in each group. The solid curve shows the average RDF from all measured nuclei and the shaded area shows the standard error. The P-value between wild-type vs. 6-week *BRAF*^{V600E} and 6-week vs. 12-month *BRAF*^{V600E} are $P < 10^{-18}$ and $P < 10^{-2}$, respectively. All *P* values were determined using Mann-Whitney test.

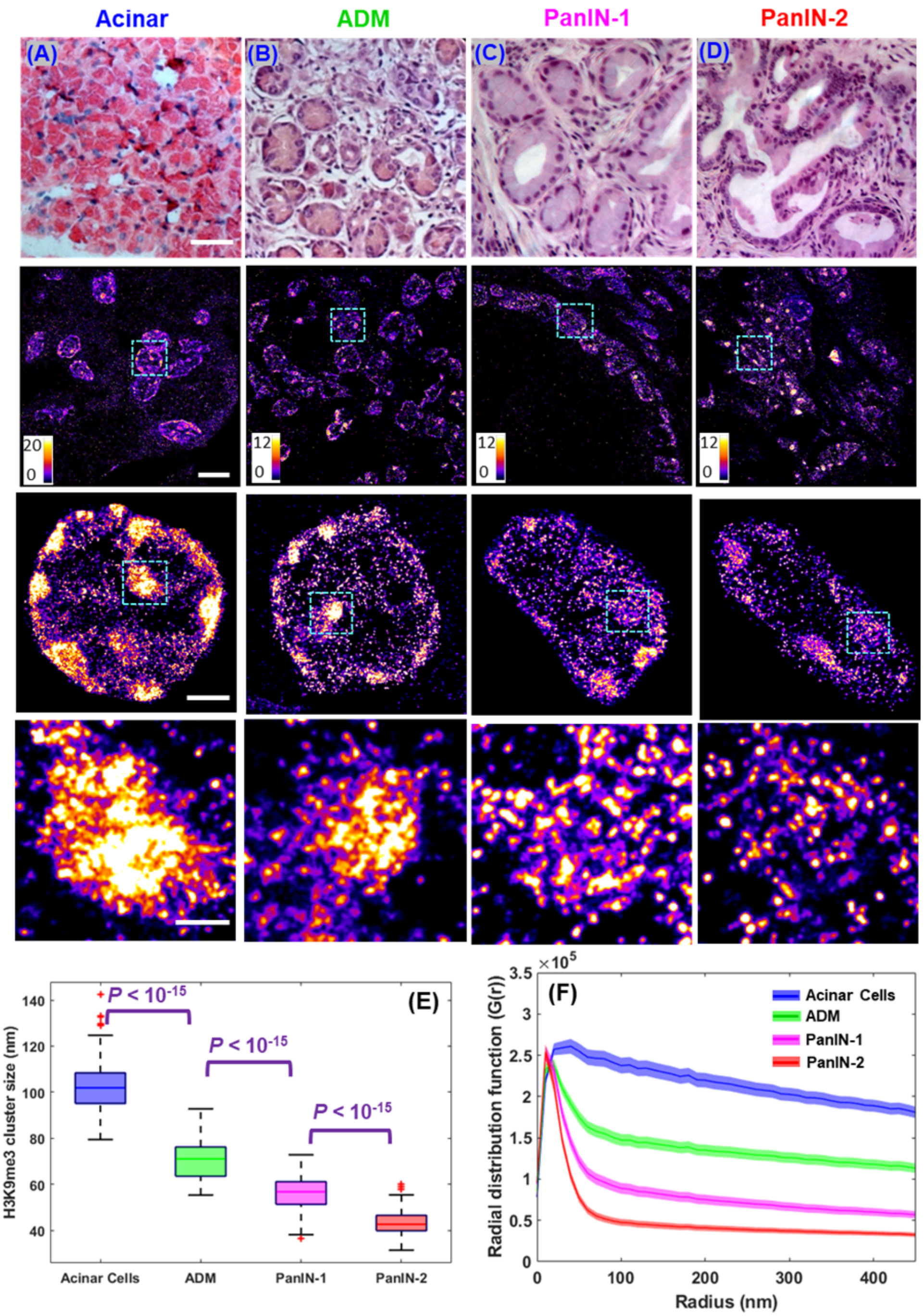

Supplementary Figure 19. (A-D) Representative histology (scale bar: 200 μm) and super-resolution images of H3K9me3-dependent heterochromatin structure from normal acinar cells of the pancreas from wild-type mice, acinar-to-ductal metaplasia (ADM), pancreatic intraepithelial lesions (PanIN) grade 1 and 2 from *Pdx1-Cre KRAS*$^{G12D/+}$ mice. The scale bars in original and magnified STORM images are 10μm, 2μm and 500 nm, respectively. (**E**) Box-and-whisker plot of the H3K9me3 cluster size (n = 115, 103, 122, 144 cells, respectively), where the central line of the box indicates the median; the bottom/top edge of the box indicate 25th/75th percentiles. (**F**) Radial distribution function (RDF) that quantifies H3K9me3-dependent heterochromatin structure, averaged over all nuclei for each group. The central solid line is the mean and the shaded area shows the standard error. The P-value between two groups (acinar cells vs. AMD, ADM vs. PanIN-1, and PanIN-1 vs. PanIN-2) are all $P < 10^{-10}$. All *P* values were determined using Mann-Whitney test.

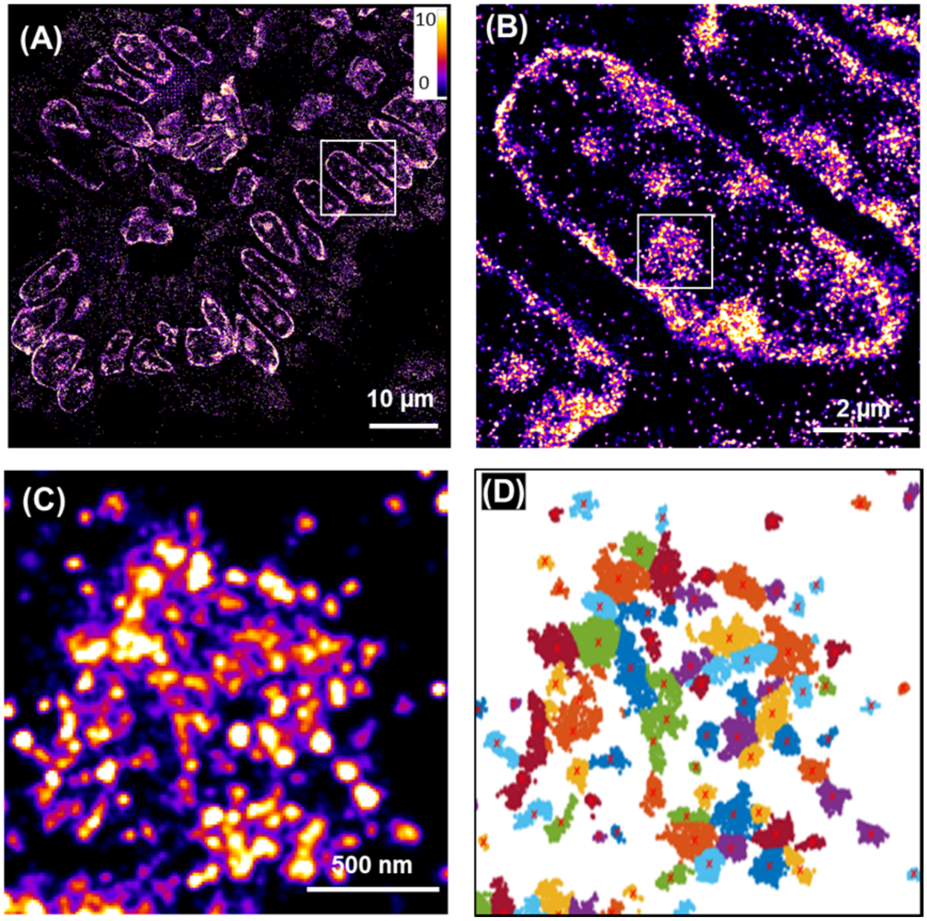

Supplementary Figure 20. Illustration of cluster analysis by Gaussian fitting. **(A)** The reconstructed STORM images. **(B)** The magnified region of the white box in (A). **(C)** The magnified region of heterochromatin foci from the white box in (B). **(D)** The identified clusters marked by different colors from the same region in (C).

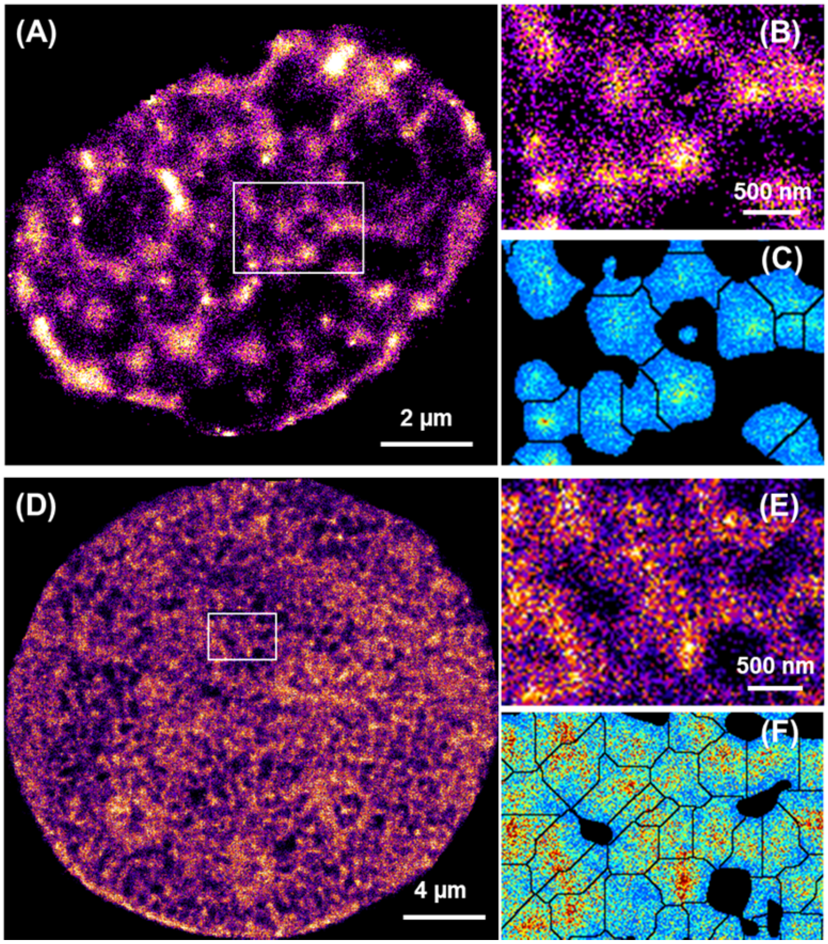

Supplementary Figure 21. Illustration of watershed-based segmentation of nanodomains within the cell nucleus. **(A, D)** The reconstructed STORM images of DNA (labeled by TOTO-3) from intestinal epithelial cells on FFPE tissue section and cultured NIH-3T3 cells. **(B, E)** The magnified region from the white box in (A, D). **(C, F)** The segmented nanodomains by watershed from the same region in (B, E).

Supplementary Table

Supplementary Table 1. Patient characteristics for human pathological tissue from surgical resection

Sample ID	Age range	Group	Pathological stage of tissue imaged by PathSTORM	Most advanced pathological stage of the patient
1	60-69	Normal	normal	Diverticulosis
2	50-59	Normal	normal	Diverticulosis
3	80-89	Normal	normal	Diverticulosis
4	80-89	Normal	normal	Diverticulosis
5	60-69	Normal	normal	Diverticulosis
6	60-69	LGD/adenoma	LGD	LGD
7	70-79	LGD/adenoma	Tubular adenoma	Adenocarcinoma
8	70-79	LGD/adenoma	Adenoma	Sissle serrated adenoma
9	70-79	LGD/adenoma	Tubular adenoma	Tubular adenoma
10	50-59	LGD/adenoma	Tubular adenoma	Tubular adenoma
11	50-59	HGD	HGD	HGD
12	80-89	HGD	HGD	Tubular adenoma with focal HGD
13*	20-29	HGD	Adenoma with HGD	Adenocarcinoma
14	60-69	HGD	HGD	Adenocarcinoma
15	50-59	Cancer	Adenocarcinoma	Adenocarcinoma
16*	20-29	Cancer	Adenocarcinoma	Adenocarcinoma
17	80-89	Cancer	Adenocarcinoma	Adenocarcinoma
18	80-89	Cancer	Adenocarcinoma	Adenocarcinoma
19	60-69	Cancer	Adenocarcinoma	Adenocarcinoma

* Sample #13 and #16 are from the same patient.

Supplementary Table 2. Resource Table

Reagent or Resource	Source	Identifier
**Antibodies**		
Rabbit polyclonal to H3K9me3	Abcam	Cat#: ab8898
Rabbit polyclonal to H3K27me3	EMD Millipore	Cat#: 07-449
Rabbit polyclonal to Histone H3	Abcam	Cat#: ab1791
Rabbit polyclonal to H3K4me3	Abcam	Cat#: ab8580
Rabbit polyclonal to H3K4me3	EMD Millipore	Cat#: 05-745R
Rabbit polyclonal to H4Ac	EMD Millipore	Cat#: 06-598
Rabbit monoclonal to β-Tubulin	Cell Signaling Technology	Cat#: 6181s
Rabbit polyclonal to KMT1A/SUV39H1	Novus Biologicals	Cat#: NBP2-17086
Mouse monoclonal to Ki67	Cell Signaling Technology	Cat#: 9449s
Mouse monoclonal to RNAP II	Abcam	Cat#: ab5408
Mouse monoclonal to γ-H2AX (Ser 139)	Santa Cruz Biotechnology	Cat#: sc-517348
Mouse monoclonal to BrdU	Cell Signaling Technology	Cat#: #5292
Donkey anti-rabbit antibody	Jackson ImmunoResearch	Cat#: 711-005-152
Donkey anti-mouse antibody	Jackson ImmunoResearch	Cat#: 715-005-151
**Chemicals and Reagent**		
proteinA-MNase, purified from pK19pA-MN	Addgene	Plasmid #86973
Suv39h1 siRNA	Integrated DNA Technologies	Customized
Click-iT Plus EdU (5-ethynyl-2′-deoxyuridine) Alexa Fluor Imaging Kit	Thermo Fisher Scientific	Cat#: C10640
TOTO™-3 Iodide	Thermo Fisher Scientific	Cat#: T3604
2,2'-thiodiethanol (TDE)	Sigma-Aldrich	Cat#: 166782
Glucose	Sigma-Aldrich	Cat#: G7021
2-mercaptoethanol (βME)	Sigma-Aldrich	Cat#: 63689
Glucose Oxidase from Aspergillus niger	Sigma-Aldrich	Cat#: G2133
Catalase from bovine liver	Sigma-Aldrich	Cat#: C40
Cyclooctatetraene (COT)	Sigma-Aldrich	Cat#: 138924
5,6-dichloro-1-β-D ribofuranosylbenzimidazole	Sigma-Aldrich	Cat#: D1916
KaryoMAX™ Giemsa Stain Solution	Thermo Fisher Scientific	Cat#: 10092013
Colcemid	Thermo Fisher Scientific	Cat#: 15212012
BrdU (5-Bromo-2′-Deoxyuridine)	Thermo Fisher Scientific	Cat#: B23151
Alexa 647 carboxylic acid succinimidyl ester	Thermo Fisher Scientific	Cat#: A20006
Cy3B reactive dye	GE Healthcare	Cat#: PA63101
CF568 Azide	Biotium	Cat#: 92082
FluoSpheres™ Carboxylate-Modified Microspheres, 0.1 µm, yellow-green fluorescent (505/515), 2% solids	Thermo Fisher Scientific	Cat#: F8803
TetraSpeckTM Microspheres, 0.1 µm, fluorescent blue/green/orange/dark red	Thermo Fisher Scientific	Cat#: T7279
TRIzol reagent for RNA extraction	Invitrogen	Cat#: 15596026
**Deposited Data**		
CUT&RUN datasets	This paper	GEO: GSE121800
RNA-seq datasets	This paper	GEO: GSE121800
**Cell Lines, Animal Models and Human Patients Samples**		

NIH/3T3 cell lines	ATCC	ATCC® CRL-1658™
Mouse: B6.129-$Kras^{tm4Tyj}$/Nci	NCI Mouse Repository	STRAIN 01XJ6
Mouse: B6.FVB-Tg(Pdx1-cre)6Tuv/Nci	NCI Mouse Repository	STRAIN 01XL5
Mouse: B6.Cg-Tg(Vil1-cre)1000Gum/J	The Jackson Laboratory	Stock No 21504
Mouse: B6.129P2(Cg)-$Braf^{tm1Mmcm}$/J	The Jackson Laboratory	Stock No 17837
Mouse: C57BL/6J-Apc^{Min}/J	The Jackson Laboratory	Stock No 002020
Mouse: C57BL/6J	The Jackson Laboratory	Stock No 000664
Mouse: FVB-Tg(ARR2/Pbsn-MYC)7Key/Nci	NCI Mouse Repository	STRAIN 01XK8
Human colon tissue	Department of Anatomic Pathology, University of Pittsburgh Medical Center	De-identified

Supplementary References

1. Xu, J. *et al.* Super-Resolution Imaging of Higher-Order Chromatin Structures at Different Epigenomic States in Single Mammalian Cells. *Cell Rep* **24**, 873-882 (2018).

2. Ma, H., Xu, J., Jin, J., Huang, Y. & Liu, Y. A Simple Marker-Assisted 3D Nanometer Drift Correction Method for Superresolution Microscopy. *Biophys J* **112**, 2196-2208 (2017).

3. Xu, J. & Liu, Y. Imaging Higher-order Chromatin Structures in Single Cells Using Stochastic Optical Reconstruction Microscopy. *Bio Protoc* **9** (2019).

4. Porto, V. *et al.* Silver Atomic Quantum Clusters of Three Atoms for Cancer Therapy: Targeting Chromatin Compaction to Increase the Therapeutic Index of Chemotherapy. *Adv. Mater* **30**, 1801317 (2018).

5. Tripathi, S. *et al.* Meta- and Orthogonal Integration of Influenza "OMICs" Data Defines a Role for UBR4 in Virus Budding. *Cell Host Microbe* **18**, 723-735 (2015).
