# Supplementary material for: Super-resolution imaging reveals the evolution of higher-order chromatin folding in early carcinogenesis
Source: Nat Commun. 2020 Apr 20;11:1899. doi: 10.1038/s41467-020-15718-7 (PMC7171144; doi:10.1038/s41467-020-15718-7)
Supplement: Supplementary file 2 — Reporting Summary [file 41467_2020_15718_MOESM2_ESM.pdf]

nature research

Corresponding author(s): Yang Liu

Last updated by author(s): Mar 18, 2020

Reporting Summary

Nature Research wishes to improve the reproducibility of the work that we publish. This form provides structure for consistency and transparency in reporting. For further information on Nature Research policies, see Authors & Referees and the Editorial Policy Checklist .

Statistics

For all statistical analyses, confirm that the following items are present in the figure legend, table legend, main text, or Methods section.

n/a	Confirmed	
☐	☒	The exact sample size (n) for each experimental group/condition, given as a discrete number and unit of measurement
☐	☒	A statement on whether measurements were taken from distinct samples or whether the same sample was measured repeatedly
☐	☒	The statistical test(s) used AND whether they are one- or two-sided<br>*Only common tests should be described solely by name; describe more complex techniques in the Methods section.*
☒	☐	A description of all covariates tested
☐	☒	A description of any assumptions or corrections, such as tests of normality and adjustment for multiple comparisons
☐	☒	A full description of the statistical parameters including central tendency (e.g. means) or other basic estimates (e.g. regression coefficient) AND variation (e.g. standard deviation) or associated estimates of uncertainty (e.g. confidence intervals)
☐	☒	For null hypothesis testing, the test statistic (e.g. F, t, r) with confidence intervals, effect sizes, degrees of freedom and P value noted<br>*Give P values as exact values whenever suitable.*
☒	☐	For Bayesian analysis, information on the choice of priors and Markov chain Monte Carlo settings
☒	☐	For hierarchical and complex designs, identification of the appropriate level for tests and full reporting of outcomes
☒	☐	Estimates of effect sizes (e.g. Cohen's d, Pearson's r), indicating how they were calculated

Our web collection on statistics for biologists contains articles on many of the points above.

Software and code

Policy information about availability of computer code

Data collection

STORM images were acquired using custom-built system on the Olympus IX71 inverted microscope.
3D-SIM images were acquired by N-SIM system (Nikon).
Western blot detection was done using BIORAD Universal Hood II machine with ImageLab software.
Size distribution of libraries was determined using a Fragment analyzer, and data was delivered via email. Libraries were sequenced on an Illumina NextSeq500. Fastq files were transferred via BaseSpace (Illumina)
Duplicates were removed using Picard version 2.18.12 (http://broadinstitute.github.io/picard/).
Reads with low quality score (MAPQ < 10) were removed using SamTools version 1.9.

Data analysis

STORM image analysis:
The background correction was performed using the same method reported in Ma et al, Sci Adv 5, eaaw0683 (2019) and the code was available on https://pitt.box.com/v/WindSTORM (password: Biophotonics).
The STORM image reconstruction was performed by custom software written in CUDA-C, available upon request.
Cluster analysis, radial distribution function (RDF), Voronoi tessellation analysis and Watershed analysis were performed with custom software written in MATLAB 2019a (Mathworks), available upon request.
Statistical analyses were performed using GraphPad Prism 8 software.

CUT&RUN analysis:
Paired-end reads were trimmed to 25 bases and barcodes were removed using Novocraft.
Reads were aligned to mm10 using Bowtie2 (version 2.3.4.2) with the parameter -X 1000.
Reads were separated into the following size classes: <120bp for TF occupancy and 150-500bp for nucleosome occupancy using the "awk" command and samtools.
Reads were processed using HOMER (http://homer.ucsd.edu/homer/index.html) commands including:
"makeUCSCfile"; "findPeaks"; "mergePeaks"; "findMotifs".
The codes for CUT&RUN analysis are the same as previously published (Hainer et al 2019 Cell), and are already available on GitHub here:

https://github.com/sarahhainer/uliCUT-RUN.

For RNAseq analysis:
Reads were aligned to mm10 using RSEM (version 1.2.29).
TPM was calculated to visualize gene expression changes.
Mapped reads were processed using HOMER (http://homer.ucsd.edu/homer/index.html) and DESeq2 (version XX) to identify differentially expressed genes.
K-means clustering was performed using Cluster 3.0 and heatmaps were generated using Java TreeView (version 1.1.6r4).
Gene Ontology term enrichment was performed using Metascape (https://metascape.org/gp/index.html#/main/step1).

For manuscripts utilizing custom algorithms or software that are central to the research but not yet described in published literature, software must be made available to editors/reviewers. We strongly encourage code deposition in a community repository (e.g. GitHub). See the Nature Research guidelines for submitting code & software for further information.

Data

Policy information about availability of data

All manuscripts must include a data availability statement. This statement should provide the following information, where applicable:

- Accession codes, unique identifiers, or web links for publicly available datasets
- A list of figures that have associated raw data
- A description of any restrictions on data availability

All CUT&RUN and RNA-seq sequencing data are deposited at Gene Expression Omnibus with accession code GSE121800. Representative raw data of STORM imaging videos are located at: https://pitt.app.box.com/v/PathSTORM-RawData. Given the large dataset, all other relevant data are available from the corresponding authors upon reasonable request.

Field-specific reporting

Please select the one below that is the best fit for your research. If you are not sure, read the appropriate sections before making your selection.

☒ Life sciences ☐ Behavioural & social sciences ☐ Ecological, evolutionary & environmental sciences

For a reference copy of the document with all sections, see nature.com/documents/nr-reporting-summary-flat.pdf

Life sciences study design

All studies must disclose on these points even when the disclosure is negative.

Sample size	The imaging experiments were performed on about 50 to 300 cell nuclei per mouse and three mice per group. The sample size was determined based on our preliminary experiments to detect a significant difference in the structural features at different stages in carcinogenesis using ANOVA and Mann-Whitney test.
Data exclusions	No data were excluded.
Replication	The imaging results were independently validated on a minimum of three biological replicate mice per group. Two to three biological replicate mice were utilized for genomic experiments, and experiments were performed independently.
Randomization	No randomization was performed. Randomization was not relevant in our study to understand the changes of chromatin structure at different stages of carcinogenesis.
Blinding	No blinding was performed since all deep sequencing dataset for each experiment were processed in parallel.

Behavioural & social sciences study design

All studies must disclose on these points even when the disclosure is negative.

Study description	*Briefly describe the study type including whether data are quantitative, qualitative, or mixed-methods (e.g. qualitative cross-sectional, quantitative experimental, mixed-methods case study).*
Research sample	*State the research sample (e.g. Harvard university undergraduates, villagers in rural India) and provide relevant demographic information (e.g. age, sex) and indicate whether the sample is representative. Provide a rationale for the study sample chosen. For studies involving existing datasets, please describe the dataset and source.*
Sampling strategy	*Describe the sampling procedure (e.g. random, snowball, stratified, convenience). Describe the statistical methods that were used to predetermine sample size OR if no sample-size calculation was performed, describe how sample sizes were chosen and provide a rationale for why these sample sizes are sufficient. For qualitative data, please indicate whether data saturation was considered, and what criteria were used to decide that no further sampling was needed.*

Data collection	*Provide details about the data collection procedure, including the instruments or devices used to record the data (e.g. pen and paper, computer, eye tracker, video or audio equipment) whether anyone was present besides the participant(s) and the researcher, and whether the researcher was blind to experimental condition and/or the study hypothesis during data collection.*
Timing	*Indicate the start and stop dates of data collection. If there is a gap between collection periods, state the dates for each sample cohort.*
Data exclusions	*If no data were excluded from the analyses, state so OR if data were excluded, provide the exact number of exclusions and the rationale behind them, indicating whether exclusion criteria were pre-established.*
Non-participation	*State how many participants dropped out/declined participation and the reason(s) given OR provide response rate OR state that no participants dropped out/declined participation.*
Randomization	*If participants were not allocated into experimental groups, state so OR describe how participants were allocated to groups, and if allocation was not random, describe how covariates were controlled.*

Ecological, evolutionary & environmental sciences study design

All studies must disclose on these points even when the disclosure is negative.

Study description	*Briefly describe the study. For quantitative data include treatment factors and interactions, design structure (e.g. factorial, nested, hierarchical), nature and number of experimental units and replicates.*
Research sample	*Describe the research sample (e.g. a group of tagged Passer domesticus, all Stenocereus thurberi within Organ Pipe Cactus National Monument), and provide a rationale for the sample choice. When relevant, describe the organism taxa, source, sex, age range and any manipulations. State what population the sample is meant to represent when applicable. For studies involving existing datasets, describe the data and its source.*
Sampling strategy	*Note the sampling procedure. Describe the statistical methods that were used to predetermine sample size OR if no sample-size calculation was performed, describe how sample sizes were chosen and provide a rationale for why these sample sizes are sufficient.*
Data collection	*Describe the data collection procedure, including who recorded the data and how.*
Timing and spatial scale	*Indicate the start and stop dates of data collection, noting the frequency and periodicity of sampling and providing a rationale for these choices. If there is a gap between collection periods, state the dates for each sample cohort. Specify the spatial scale from which the data are taken*
Data exclusions	*If no data were excluded from the analyses, state so OR if data were excluded, describe the exclusions and the rationale behind them, indicating whether exclusion criteria were pre-established.*
Reproducibility	*Describe the measures taken to verify the reproducibility of experimental findings. For each experiment, note whether any attempts to repeat the experiment failed OR state that all attempts to repeat the experiment were successful.*
Randomization	*Describe how samples/organisms/participants were allocated into groups. If allocation was not random, describe how covariates were controlled. If this is not relevant to your study, explain why.*
Blinding	*Describe the extent of blinding used during data acquisition and analysis. If blinding was not possible, describe why OR explain why blinding was not relevant to your study.*

Did the study involve field work? ☐ Yes ☐ No

Field work, collection and transport

Field conditions	*Describe the study conditions for field work, providing relevant parameters (e.g. temperature, rainfall).*
Location	*State the location of the sampling or experiment, providing relevant parameters (e.g. latitude and longitude, elevation, water depth).*
Access and import/export	*Describe the efforts you have made to access habitats and to collect and import/export your samples in a responsible manner and in compliance with local, national and international laws, noting any permits that were obtained (give the name of the issuing authority, the date of issue, and any identifying information).*
Disturbance	*Describe any disturbance caused by the study and how it was minimized.*

Reporting for specific materials, systems and methods

We require information from authors about some types of materials, experimental systems and methods used in many studies. Here, indicate whether each material, system or method listed is relevant to your study. If you are not sure if a list item applies to your research, read the appropriate section before selecting a response.

Materials & experimental systems

n/a	Involved in the study
☐	☒ Antibodies
☐	☒ Eukaryotic cell lines
☒	☐ Palaeontology
☐	☒ Animals and other organisms
☐	☒ Human research participants
☒	☐ Clinical data

Methods

n/a	Involved in the study
☐	☒ ChIP-seq
☒	☐ Flow cytometry
☒	☐ MRI-based neuroimaging

Antibodies

Antibodies used

Antibodies used for CUT&RUN were H3K9me3 (abcam ab8898), H3K4me3 (Millipore 05-745R), and total H3 (abcam ab1791). Other antibodies used in the imaging study were described in Resource table in Supplementary Materials.

Validation

For H3K9me3 validation (from manufacturer): Histone H3 (tri methyl K9) antibody (ab8898) is specific for Histone H3 tri methyl Lysine 9. Shows slight cross-reactivity with tri methyl K27, which shares a similar epitope (please see Western blot image). Does not react with mono or di-methylated K9. Validated in ChIP experiments. Positive control: ChIP coupled with a peptide competition assay to validate the specificity of the antibody. Negative control: Genomic region (chr10:79154149-79155200) with no evidence of H3K9me3.

For H3K4me3 validation (from manufacturer): Chromatin immunoprecipitation was performed using the Magna ChIP™ HiSens kit (cat# 17-10460), 3 µL of Anti-trimethyl-Histone H3 (Lys4) antibody (cat# 05-745R), 20 µL Protein A/G beads, and 1e6 crosslinked HeLa cell chromatin followed by DNA purification using magnetic beads. Libraries were prepared from Input and ChIP DNA samples using standard protocols with Illumina barcoded adapters, and analyzed on Illumina HiSeq instrument. The highest 25% of peaks identified in the 05-745R and 07-473 datasets showed 99% overlap with peaks identified in the ENCODE H3K4me3 BROAD Histone track for HeLa S3.

For H3 antibody validation (from the manufacturer): Chromatin from Xenopus laevis oocytes was prepared according to the Abcam X-ChIP protocol. Oocytes were fixed with formaldehyde for 10 minutes. The ChIP was performed with 25 mg of chromatin, 3 mg of ab7834 (anti-H3, light blue) and 3 µg of ab1791 (anti-H3, dark blue), and 20 ml of Protein A/G sepharose beads. A non-specific antibody was used as a control (yellow). The immunoprecipitated DNA was quantified by real time PCR (Taqman approach).

For H3K27me3 validation (from the manufacturer): Anti-trimethyl-Histone H3 (Lys27), also known as Anti-H3K27me3, is a highly published Rabbit Polyclonal Antibody. This protein A purified antibody is dot blot tested for trimethylated lysine 27 specificity and validated in WB, ICC, IP.

For H4ac antibody validation (from the manufacturer): Anti-acetyl-Histone H4 Antibody is a Rabbit Polyclonal Antibody for detection of acetyl-Histone H4 also known as H4 histone family member A, histone 1-H4a & has been validated in ChIP, ICC, IP & WB. Cross-reacts with acetylated histone H2B fromTetrahymena and weakly cross-reacts with acetylated histone H2B from HeLa cells. May crossreact with other acetylated proteins.

For β-Tubulin antibody validation (from the manufacturer): β-Tubulin (9F3) Rabbit mAb (Biotinylated) detects endogenous levels of total β-tubulin protein, and does not cross-react with recombinant α-tubulin.

For KMT1A/SUV39H1 antibody validation (from the manufacturer): Recombinant protein encompassing a sequence within the center region of human SUV39H1. The exact sequence is proprietary. Gene ID 6839.

For Ki67 antibody validation (from the manufacturer): Ki-67 (8D5) Mouse mAb recognizes endogenous levels of total Ki-67 protein. UniProt ID: P46013, Entrez-Gene Id: 4288.

For RNAP II antibody validation (from the manufacturer): Mouse monoclonal [4H8] to RNA polymerase II CTD repeat YSPTSPS (phospho S5) - ChIP Grade. ELISA and peptide blocking show that ab5408 preferentially binds phospho S5 RNA polymerase II CTD repeat YSPTSPS. Synthetic peptide corresponding to Human RNA polymerase II CTD repeat YSPTSPS (phospho S5). The sequence is repeated multiple times in the C-terminal domain of RNA polymerase II. Database link: P24928.

For γ-H2AX antibody validation (from the manufacturer): p-Histone H2A.X Antibody (Ser 139) is a mouse monoclonal IgG1, raised against a recombinant protein corresponding to the Ser 139 phosphorylated region of Histone H2A.X of human origin recommended for detection of Ser 139 phosphorylated Histone H2A.X of mouse, rat and human origin by WB, IP and IF. This antibody has been validated by 17 publications listed on the manufacturer's website.

For BrdU antibody validation (from the manufacturer): BrdU (Bu20a) Mouse mAb detects BrdU when incorporated into single stranded DNA. the antibody was validated by the manufacturer in-house, multiple cell types, multiple methods, and specific controls were used to verify that the product will generate biologically relevant results. This antibody was also validated by 67 publications listed on the manufacturer's website.

Eukaryotic cell lines

Policy information about cell lines

Cell line source(s)	NIH 3T3 cell line were purchased from ATCC.
Authentication	The cell line was directly purchased from ATCC who has performed authentication. Below are cited from the ATCC website "Cell lines from ATCC have been thoroughly tested and authenticated, so you can be certain of their identity. ATCC uses morphology, karyotyping, and PCR based approaches to confirm the identity of human cell lines and to rule out both intra- and interspecies contamination. These include an assay to detect species specific variants of the cytochrome C oxidase I gene (COI analysis) to rule out inter-species contamination and short tandem repeat (STR) profiling to distinguish between individual human cell lines and rule out intra-species contamination"
Mycoplasma contamination	Cells were tested for mycoplasma negative.
Commonly misidentified lines (See ICLAC register)	None.

Palaeontology

Specimen provenance	*Provide provenance information for specimens and describe permits that were obtained for the work (including the name of the issuing authority, the date of issue, and any identifying information).*
Specimen deposition	*Indicate where the specimens have been deposited to permit free access by other researchers.*
Dating methods	*If new dates are provided, describe how they were obtained (e.g. collection, storage, sample pretreatment and measurement), where they were obtained (i.e. lab name), the calibration program and the protocol for quality assurance OR state that no new dates are provided.*

☐ Tick this box to confirm that the raw and calibrated dates are available in the paper or in Supplementary Information.

Animals and other organisms

Policy information about studies involving animals; ARRIVE guidelines recommended for reporting animal research

Laboratory animals	Mouse: B6.129-Krastm4Tyj/Nci, NCI Mouse Repository, STRAIN 01XJ6, Male<br>Mouse: B6.FVB-Tg(Pdx1-cre)6Tuv/Nci, NCI Mouse Repository, STRAIN 01XL5, Male<br>Mouse: B6.Cg-Tg(Vil1-cre)1000Gum/J, The Jackson Laboratory, Stock No 21504, Male<br>Mouse: B6.129P2(Cg)-Braftm1Mmcm/J,The Jackson Laboratory, Stock No 17837, Male<br>Mouse: C57BL/6J-ApcMin/J, The Jackson Laboratory, Stock No 002020, Male<br>Mouse: C57BL/6J,The Jackson Laboratory, Stock No 000664, Male<br>Mouse: FVB-Tg(ARR2/Pbsn-MYC)7Key/Nci, NCI Mouse Repository STRAIN 01XK8, Male<br>Mice were sacrificed at different ages per the experiments described in Methods and Results, and Supplementary Methods.
Wild animals	This study did not involve wild animals.
Field-collected samples	This study did not involve samples collected from the field.
Ethics oversight	All animal studies were performed in accordance with the institutional Animal Care and Use Committee of the University of Pittsburgh.

Note that full information on the approval of the study protocol must also be provided in the manuscript.

Human research participants

Policy information about studies involving human research participants

Population characteristics	Patient characteristics were described in Supplementary Table 1.
Recruitment	Archived human tissue blocks from de-identified patients were used, and the tissue was collected as the part of the standard clinical care.
Ethics oversight	The study was approved by Institutional Review Board at University of Pittsburgh.

Note that full information on the approval of the study protocol must also be provided in the manuscript.

ChIP-seq

Data deposition

- [x] Confirm that both raw and final processed data have been deposited in a public database such as GEO.
- [x] Confirm that you have deposited or provided access to graph files (e.g. BED files) for the called peaks.

Data access links<br>*May remain private before publication.*	CUT&RUN data is deposited on GEO (GSE121800). Reviewer access code: yrgfuqugpbghjmp
Files in database submission	WT_NoAb_rep1_R1.fq.gz<br>WT_NoAb_rep1_R2.fq.gz<br>WT_NoAb_rep2_R1.fq.gz<br>WT_NoAb_rep2_R2.fq.gz<br>APC_NoAb_rep1_R1.fq.gz<br>APC_NoAb_rep1_R2.fq.gz<br>APC_NoAb_rep2_R1.fq.gz<br>APC_NoAb_rep2_R2.fq.gz<br>WT_H3_rep1_R1.fq.gz<br>WT_H3_rep1_R2.fq.gz<br>WT_H3_rep2_R1.fq.gz<br>WT_H3_rep2_R2.fq.gz<br>APC_H3_rep1_R1.fq.gz<br>APC_H3_rep1_R2.fq.gz<br>APC_H3_rep2_R1.fq.gz<br>APC_H3_rep2_R2.fq.gz<br>WT_H3K9me3_rep1_R1.fq.gz<br>WT_H3K9me3_rep1_R2.fq.gz<br>WT_H3K9me3_rep2_R1.fq.gz<br>WT_H3K9me3_rep2_R2.fq.gz<br>APC_H3K9me3_rep1_R1.fq.gz<br>APC_H3K9me3_rep1_R2.fq.gz<br>APC_H3K9me3_rep2_R1.fq.gz<br>APC_H3K9me3_rep2_R2.fq.gz<br>WT_H3K4me3_rep1_R1.fq.gz<br>WT_H3K4me3_rep1_R2.fq.gz<br>WT_H3K4me3_rep2_R1.fq.gz<br>WT_H3K4me3_rep2_R2.fq.gz<br>APC_H3K4me3_rep1_R1.fq.gz<br>APC_H3K4me3_rep1_R2.fq.gz<br>APC_H3K4me3_rep2_R1.fq.gz<br>APC_H3K4me3_rep2_R2.fq.gz<br><br>WT_NoAb_rep1_150-500.ucsc.bedGraph.gz<br>WT_NoAb_rep2_150-500.ucsc.bedGraph.gz<br>APC_NoAb_rep1_150-500.ucsc.bedGraph.gz<br>APC_NoAb_rep2_150-500.ucsc.bedGraph.gz<br>WT_H3_rep1_150-500.ucsc.bedGraph.gz<br>WT_H3_rep2_150-500.ucsc.bedGraph.gz<br>APC_H3_rep1_150-500.ucsc.bedGraph.gz<br>APC_H3_rep2_150-500.ucsc.bedGraph.gz<br>WT_H3K9me3_rep1_150-500.ucsc.bedGraph.gz<br>WT_H3K9me3_rep2_150-500.ucsc.bedGraph.gz<br>APC_H3K9me3_rep1_150-500.ucsc.bedGraph.gz<br>APC_H3K9me3_rep2_150-500.ucsc.bedGraph.gz<br>WT_H3K4me3_rep1_150-500.ucsc.bedGraph.gz<br>WT_H3K4me3_rep2_150-500.ucsc.bedGraph.gz<br>APC_H3K4me3_rep1_150-500.ucsc.bedGraph.gz<br>APC_H3K4me3_rep2_150-500.ucsc.bedGraph.gz
Genome browser session<br>(e.g. UCSC)	n/a

Methodology

Replicates	CUT&RUN experiments were performed twice for each antibody on samples from both WT or APC mice.

Sequencing depth

All CUT&RUN experiments are paired-end reads (PE50), trimmed to 21 bases.
Number of reads followed by uniquely mapped reads to mm10 genome for each sample are as follows:

WT_NoAb_rep1: 517908
WT_NoAb_rep2: 2205007
APC_NoAb_rep1: 81388
APC_NoAb_rep2: 42291
WT_H3_rep1: 4699950
WT_H3_rep2: 14036681
APC_H3_rep1: 9317887
APC_H3_rep2: 15286598
WT_H3K9me3_rep1: 5412180
WT_H3K9me3_rep2: 10555789
APC_H3K9me3_rep1: 4196229
APC_H3K9me3_rep2: 8253052
WT_H3K4me3_rep1: 5694707
WT_H3K4me3_rep2: 13760358
APC_H3K4me3_rep1: 6837591
APC_H3K4me3_rep2: 16967730

Antibodies

H3K9me3, abcam Cat #ab8898, Lot #GR3217595-1
H3K4me3, Millipore Cat #05-745R, Lot #2918042
Total H3, abcam Cat #ab1791, Lot #GR3198215-1

Peak calling parameters

Reads were aligned to mm10 using Bowtie2 with the parameter -X 1000.
Peaks were called using the "findPeaks" command in HOMER (http://homer.ucsd.edu/homer/index.html).
Controls were no primary antibody (referred to throughout as "No Antibody") paired with each experiment.

Data quality

Mapping of CUT&RUN data is compared to previously published ChIP-seq data throughout.
Peak calling for CUT&RUN data is performed using HOMER "findPeak" commands. The control is the "No Antibody" experiment, paired with each experimental condition.
HOMER uses two parameters to filter peaks against a control experiment. First, it uses a fold change (which is sequencing depth-independent), requiring each putative peak to have 5-fold more normalized tags in the target experiment than the control. In the case where there are no input tags near the putative peak, HOMER automatically sets these regions to be set to the average input tag coverage to avoid dividing by zero. HOMER also uses the poisson distribution to determine the chance that the differences in tag counts are statistically significant (sequencing-depth dependent), requiring a cumulative poisson p-value of 0.0001. This effectively removes peaks with low tag counts for which there is a chance the differential enrichment is found simply due to sampling error.

HOMER assumes the local density of tags follows a Poisson distribution, and uses this to estimate the expected peak numbers given the input parameters much more quickly. Using the expected distribution of peaks, HOMER calculates the expected number of false positives in the data set for each tag threshold, setting the threshold that beats the desired False Discovery Rate, automatically set to 5%.

Software

Reads were aligned to mm10 using Bowtie2 with the parameter -X 1000
Unique reads were identified using Picard.
Reads were separated into the following size classes: <120bp for TF occupancy and 150-500bp for nucleosome occupancy using the "awk" command and samtools.
Reads were processed using HOMER (http://homer.ucsd.edu/homer/index.html) commands including: "makeUCSCfile"; "findPeaks"; "mergePeaks"; "findMotifs"

Flow Cytometry

Plots

Confirm that:

☐ The axis labels state the marker and fluorochrome used (e.g. CD4-FITC).

☐ The axis scales are clearly visible. Include numbers along axes only for bottom left plot of group (a 'group' is an analysis of identical markers).

☐ All plots are contour plots with outliers or pseudocolor plots.

☐ A numerical value for number of cells or percentage (with statistics) is provided.

Methodology

Sample preparation

Describe the sample preparation, detailing the biological source of the cells and any tissue processing steps used.

Instrument

Identify the instrument used for data collection, specifying make and model number.

Software	*Describe the software used to collect and analyze the flow cytometry data. For custom code that has been deposited into a community repository, provide accession details.*
Cell population abundance	*Describe the abundance of the relevant cell populations within post-sort fractions, providing details on the purity of the samples and how it was determined.*
Gating strategy	*Describe the gating strategy used for all relevant experiments, specifying the preliminary FSC/SSC gates of the starting cell population, indicating where boundaries between "positive" and "negative" staining cell populations are defined.*

☐ Tick this box to confirm that a figure exemplifying the gating strategy is provided in the Supplementary Information.

Magnetic resonance imaging

Experimental design

Design type	*Indicate task or resting state; event-related or block design.*
Design specifications	*Specify the number of blocks, trials or experimental units per session and/or subject, and specify the length of each trial or block (if trials are blocked) and interval between trials.*
Behavioral performance measures	*State number and/or type of variables recorded (e.g. correct button press, response time) and what statistics were used to establish that the subjects were performing the task as expected (e.g. mean, range, and/or standard deviation across subjects).*

Acquisition

Imaging type(s)	*Specify: functional, structural, diffusion, perfusion.*
Field strength	*Specify in Tesla*
Sequence & imaging parameters	*Specify the pulse sequence type (gradient echo, spin echo, etc.), imaging type (EPI, spiral, etc.), field of view, matrix size, slice thickness, orientation and TE/TR/flip angle.*
Area of acquisition	*State whether a whole brain scan was used OR define the area of acquisition, describing how the region was determined.*

Diffusion MRI ☐ Used ☐ Not used

Preprocessing

Preprocessing software	*Provide detail on software version and revision number and on specific parameters (model/functions, brain extraction, segmentation, smoothing kernel size, etc.).*
Normalization	*If data were normalized/standardized, describe the approach(es): specify linear or non-linear and define image types used for transformation OR indicate that data were not normalized and explain rationale for lack of normalization.*
Normalization template	*Describe the template used for normalization/transformation, specifying subject space or group standardized space (e.g. original Talairach, MNI305, ICBM152) OR indicate that the data were not normalized.*
Noise and artifact removal	*Describe your procedure(s) for artifact and structured noise removal, specifying motion parameters, tissue signals and physiological signals (heart rate, respiration).*
Volume censoring	*Define your software and/or method and criteria for volume censoring, and state the extent of such censoring.*

Statistical modeling & inference

Model type and settings	*Specify type (mass univariate, multivariate, RSA, predictive, etc.) and describe essential details of the model at the first and second levels (e.g. fixed, random or mixed effects; drift or auto-correlation).*
Effect(s) tested	*Define precise effect in terms of the task or stimulus conditions instead of psychological concepts and indicate whether ANOVA or factorial designs were used.*

Specify type of analysis: ☐ Whole brain ☐ ROI-based ☐ Both

Statistic type for inference<br>(See Eklund et al. 2016)	*Specify voxel-wise or cluster-wise and report all relevant parameters for cluster-wise methods.*
Correction	*Describe the type of correction and how it is obtained for multiple comparisons (e.g. FWE, FDR, permutation or Monte Carlo).*

Models & analysis

n/a	Involved in the study
☒	☐ Functional and/or effective connectivity
☒	☐ Graph analysis
☒	☐ Multivariate modeling or predictive analysis
