# Supplementary figures and images for: Super-resolution imaging reveals the evolution of higher-order chromatin folding in early carcinogenesis
Source: Nat Commun. 2020 Apr 20;11:1899. doi: 10.1038/s41467-020-15718-7 (PMC7171144; doi:10.1038/s41467-020-15718-7)

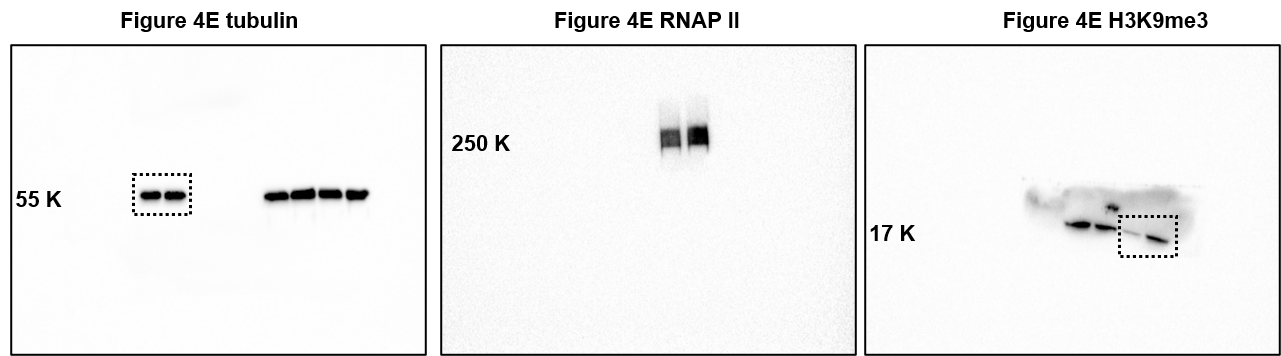

Supplement: Supplementary file 3 — Source Data [file 41467_2020_15718_MOESM3_ESM.zip › TIF.tif]
